# Targeted Nanodelivery of WGX50 and Curcumin via Gold Nanoparticles for Alzheimer's Therapy

**DOI:** 10.1111/jcmm.71045

**Published:** 2026-02-06

**Authors:** Madeeha Shahzad Lodhi, Muhammad Maisam, Muhammad Tahir Khan, Amina Bibi, Dongqing Wei, Kejie Mou

**Affiliations:** ^1^ Institute of Molecular Biology and Biotechnology The University of Lahore Lahore Pakistan; ^2^ Graduate Institute of Biomedical Science China Medical University Taichung Taiwan; ^3^ International Center for Interdisciplinary Research in Sciences (ICIRS) The University of Lahore Lahore Pakistan; ^4^ State Key Laboratory of Respiratory Disease, Guangzhou Key Laboratory of Tuberculosis Research, Department of Clinical Laboratory, Guangzhou Chest Hospital, Institute of Tuberculosis Guangzhou Medical University Guangzhou China; ^5^ State Key Laboratory of Microbial Metabolism, Joint International Research Laboratory of Metabolic & Developmental Sciences and School of Life Sciences and Biotechnology Shanghai Jiao Tong University Shanghai P.R. China; ^6^ Zhongjing Research and Industrialization Institute of Chinese Medicine Nanyang Henan PR China; ^7^ Qihe Laboratory Qishui Guang East Hebi Henan China; ^8^ Department of Neurosurgery Bishan Hospital of Chongqing Medical University Chongqing China

**Keywords:** Alzheimer's disease, curcumin, gold nanoparticles, insulin, miRNA‐146a‐5p, targeted drug delivery, WGX50

## Abstract

Alzheimer's disease (AD) is a progressive neurodegenerative disorder, posing a global health challenge. It affects millions of people, causing cognitive decline and a heavy burden on healthcare systems. Neuroinflammation is a key pathological feature of AD, often associated with the dysregulation of microRNAs such as hsa‐miR‐146a‐5p. WGX50 (N‐[2‐(3,4‐Dimethoxy‐phenyl)‐ethyl]‐3‐phenyl‐acrylamide), a small molecule derived from *Zanthoxylum bungeanum* Maxim, has antioxidant and anti‐inflammatory activities. While WGX50 demonstrates potent inhibition of neuroinflammation, its poor blood–brain barrier permeability may be improved using targeted delivery strategies. The current study aimed to design a novel nanoconjugate of WGX50 and curcumin with gold nanoparticles (AuNPs) to observe its therapeutic effects in a rat model. All nanoconjugates were synthesised as targeted (Cys‐capped AuNPs with WGX50‐insulin and curcumin‐insulin) and non‐targeted (without insulin). Immunohistochemical analysis revealed that both non‐targeted (WGX50‐NT) and targeted (WGX50‐T) therapies have a significant effect in the rat model, with WGX50‐T showing a more pronounced effect. The histopathology results of WGX50 and WGX50‐T showed an approximate 80%–90% reduction in Aβ plaque deposition. The treatment with both curcumins targeted (C‐T) and non‐targeted (C‐NT) formulations led to a significant reduction in Aβ levels in AD rats. Fluorescence microscopy confirmed that targeted delivery was more effective, potentially leading to better therapeutic outcomes. The expression levels of hsa‐miR‐146a‐5p showed differential expression levels with targeted treatments correlating with lower expression levels, suggesting a role in modulating neuroinflammation and immune responses. Overall, these findings highlight the potential of targeted drug delivery systems in enhancing the efficacy of AD treatments.

## 1 Introduction

Alzheimer's disease (AD) is a progressive neurodegenerative disorder, posing a global health challenge. It affects millions of people, causing cognitive decline and a heavy burden on healthcare systems. Plaque buildup in the hippocampus is a hallmark of AD, a slow‐moving, degenerative brain illness [1, 2]. It is challenging to pinpoint the precise course of AD disorders since plaque development starts around 20 years earlier than clinical symptoms appear. Globally, the prevalence of AD is growing, and in 2019, over 50 million individuals were afflicted ([3] Dementia Forecasting Collaborators, 2022; [2, 4]).

AD is indicated by behavioural changes, diminished memory, and cognitive impairments [5]. Neurodegeneration, which starts with synaptic degradation and advances to neuronal death, is associated with dementia [6]. The adult neurogenesis mechanism in the hippocampus may be hindered by additional elements of the neurodegenerative process [7, 8]. Cognitive dysfunction in AD patients is associated with the breakdown of synapses in limbic and neocortical circuits [9].

Extracellular amyloid plaques and intracellular tau neurofibrillary tangles (NFTs) are the two main histopathologic lesions associated with AD [10]. β‐amyloid (Aβ) cleaves to form an insoluble, proteolysis‐resistant peptide fibril, which is the primary constituent of amyloid or senile plaques (SPs). Aβ‐peptides are produced when the two enzymes β‐secretase (BACE1) and γ‐secretase successively break apart the amyloid precursor protein (APP) [11]. The complex interplay of genetic and environmental factors is an influencer of the late onset of AD, with around 70% of AD risk attributed to genetic factors. The main risk factor for sporadic AD is the *APOE* gene variants, particularly ε4 [12].

The miRNAs are small, soluble, amphipathic regulatory molecules that play a significant role in posttranscriptional and epigenetic regulation of messenger RNA (mRNA) abundance, spatiotemporal expression, and complexity. Specific miRNAs underexpressed and overexpressed are associated with physiological effects accompanying the disease, influencing aging, mitochondrial impairment, tau accumulation, and the regulation of enzymes associated with Aβ production [10]. Changes in miRNA expression contribute to alterations in gene profiles, making circulating miRNAs potential biomarkers for AD identification and treatment [13]. Despite ongoing studies, treatments for AD are still limited. Key contributors to AD include Aβ deposition, tau protein hyperphosphorylation, neurofibrillary tangles, mitochondrial dysfunction, and inflammation. Mechanistic exploration reveals their correlation with autophagy dysfunction. Neuroinflammation, characterised by the involvement of the inflammatory transcription factor NF‐κB, further exacerbates AD progression, alongside Aβ plaques and neurofibrillary tangles [14]. Within the central nervous system (CNS), a subgroup of possibly pathogenic miRNAs is found, including NF‐kB‐delicate miRNA‐146a‐5p.

The expression of miRNA differs in various neurological disorders [15], with crucial and determinant functions in the onset and progression of numerous neurological diseases. These particular miRNAs: (i) show predominant expression in the cytoplasm of brain, retinal, and other central nervous system cells and (ii) demonstrate inducibility by various pathological factors, including pro‐inflammatory cytokines and chemokines. Studies show that NF‐kB‐delicate miRNA‐146a‐5p controls the transcription activity of a particular group of brain‐specific miRNAs [16]. Human hippocampal tissue and human neocortical tissue contain moderate levels of miRNA‐146a. The miRNA‐146a expression increases at pathological levels when the brain faces pathological stimuli that include stress, together with LPS and localized ROS and Aβ42 peptide or prion amyloid accumulations. The amyloidogenic proteins function as pathological indicators of AD and prion disease separately [15].

Curcumin has anti‐inflammatory, anti‐oxidative, and anti‐amyloidogenic properties that may help prevent AD [17]. The anti‐amyloidogenic properties of curcumin and its analog, rosmarinic acid, destabilise preformed Aβ fibrils to regenerate Aβ monomers and inhibit the elongation of neurotoxic Aβ fibrils from fresh Aβ in a dose‐dependent manner [18]. Its principal impact on Aβ fibrilization makes it a promising medicine for use in treating and/or preventing AD. Despite its potent anti‐amyloidogenic properties, curcumin has some limitations that hinder its application in AD. First, curcumin exhibits very poor oral absorption, undergoes rapid metabolism and elimination, and its brain uptake is highly limited [19]. To overcome these challenges, a novel delivery system could be introduced to enhance curcumin's brain bioavailability and stability [19].

WGX50 (N‐[2‐(3,4‐Dimethoxy‐phenyl)‐ethyl]‐3‐phenyl‐acrylamide), a small molecule derived from *Zanthoxylum bungeanum* Maxim, has antioxidant and anti‐inflammatory activities [20, 21]. WGX50 inhibits neuroinflammation‐induced amyloid‐beta (Aβ) peptide via the PI3K/AKT and JAK2/STAT3 pathways. However, it exhibited poor water solubility and blood–brain barrier crossing.

To enhance WGX50 and curcumin delivery to the brain, it was conjugated with gold nanoparticles (AuNPs) and insulin. In comparison, curcumin and AuNPs were also conjugated in targeted and non‐targeted ways. Curcumin will offer a therapeutic synergy with WGX50. AuNPs significantly enhance drug delivery across the blood–brain barrier (BBB) due to their unique size and surface chemistry. AuNPs can be functionalized to increase biocompatibility and facilitate targeted delivery to specific brain regions, thereby overcoming the limitations of WGX50's poor solubility and improving its therapeutic impact [22]. N‐[2‐(3,4‐dimethoxyphenyl)ethyl]‐N‐[2‐(3,4‐dimethoxyphenyl)ethyl]‐3‐phenylacrylamide, isolated from Sichuan pepper (Zanthoxylum bungeanum) and known as WGX50, has been identified as a viable medicine for the treatment of AD [21]. However, it poorly crosses the blood–brain barrier. Therefore, the current investigation was designed to determine if the WGX50 concentration could be increased in the brain if conjugated with AuNPs and insulin in a targeted approach.

## 2 Materials and Methods

### 2.1 AuNPs Synthesis

Chemically AuNPs were prepared using the Turkevich method. The standard method, as described by Turkevich and Frens, involves citrate reduction at 100°C. A 12.5 mL of 25 mM gold hydrochlorate solution was reduced by 12.5 mL of 1% sodium tri‐citrate by bringing the gold solution to a boil in a beaker on the hotplate. No refluxing was used to avoid the presence of temperature gradients in the liquid. About 12.5 mL of warmed 1% sodium tri‐citrate solution was added when the solution began to boil. To produce particles of different sizes, the citrate content was adjusted. The liquid was extracted and cooled to room temperature after 15 min, at which point the solution turned brick red. Those synthesised AuNPs were then characterised as described in Section 3.

### 2.2 Capping of AuNPs With Linker

Cysteine was used as a linker on chemically prepared NPs. About 49.95 M of cysteine solution was prepared, and a pH of 7.4 was adjusted. Approximately 2.5 mL of cysteine was added to the chemically synthesised AuNPs and was incubated at 37°C in the dark for 2 h with constant shaking at 100 rpm, followed by an additional 24 h without shaking. Those synthesised AuNPs were then characterised as described in Section 2.8. AuNPs were capped with cysteine to conjugate carboxyl, carbonyl, and the hydroxyl group of plant phytochemicals.

### 2.3 Non‐Target Nano Formulations

Approximately 0.1 M sodium carbonate coupling buffer with a pH of 9.5 was prepared. The particles were conjugated with a concentration of 4.28 μg/μL of Cys‐AuNPs and curcumin. Curcumin was suspended at 5% w/v as a Tetrahydrofuran (THF) suspension containing Carbonyl Diimidazole (CDI) at 50 mg/mL and mixed for 2 h at room temperature. Activated particles were washed with THF to remove excess CDI and byproducts. They were resuspended at 10 mg/mL in cold coupling buffer after being washed with ice‐cold deionised water to remove any solvent. Then 1 to 10 mg of amine‐containing NPs were added and reacted by mixing for at least 18 h at 4°C. 0.1 M ethanolamine was added and mixed for 2 h. Suspended the particles in 0.1 M sodium carbonate after being centrifuged and washed three times with coupling buffer.

### 2.4 Targeted Nano Formulations

All targeted nanoformulation of WGX50 and curcumin have been synthesised using previous study protocol [23]. Briefly Cys‐AuNPs loaded with insulin, were synthesised via the polyelectrolyte complexation (PEC). Cys was dissolved in 1% (v/v) acetic acid (pH adjusted to 5.5 ± 0.1), and insulin was dissolved in 0.01 N HCl (pH adjusted to 8.0 ± 0.1). Both solutions were stirred at room temperature.

### 2.5 Characterizations

All synthesised nanoformulations were characterised using UV–Visible spectrometry, FTIR (Fourier transform infrared spectroscopy), DLS (Dynamic light scattering), Zeta potential, XRD (X‐ray diffraction), scanning electron microscopy (SEM), and Energy Dispersive X‐ray spectroscopy (EDX).

#### 2.5.1 UV–Visible Spectroscopy

It is a quantitative technique used to measure the intensity of light that passes through the sample compared to the intensity that passes through the blank. The spectrophotometer used for the current nanocomposites analysis was BIOBASE, BK‐UV1900PC.

#### 2.5.2 The FTIR Spectroscopy

This technique is also called infrared spectroscopy. It was used to identify organic, inorganic, and polymeric materials when infrared light passes through the test sample and to measure chemical properties. The infrared spectra reveal various functional groups, providing information on the chemical composition and physical state of the test samples.

#### 2.5.3 The DLS Spectroscopy

The size of all nanoconjugates was measured using DLS, which assesses how much light is reflected by the solution's particles. As the particles travel away from their starting positions due to Brownian motion, the reflected light will alter over time. Depending on the particles' hydrodynamic radius, this occurs at varying rates. As a result, the size of the suspended particles can be determined by measuring the rate of change of the reflected light. A graph of the intensity of the reflected light over the size of the particles is used to display the DLS findings.

#### 2.5.4 XRD Technique

The XRD technique was used to determine the crystalline nature of all nanoconjugates based on Bragg's law (*n* = 2dsin). For characterising crystalline materials, XRD is a widespread and non‐discriminatory approach that examines data on the structural properties of nanocomposites, including preferred crystal orientations (texture), phase purity, and other structural metrics, such as average grain size, crystallinity, strain, and crystal defects. The peaks are generated by constructive interference when a monochromatic beam of X‐rays is spread at specific angles from each pair of lattice planes in a sample. Peak intensities are based on atomic positions inside the lattice planes. A powder sample is required for XRD analysis to detect unknown crystalline compounds in geology, materials science, environmental sciences, and engineering.

#### 2.5.5 SEM Microscopy

This technique was used to provide detailed morphological information at the nanoscale. The electron beam scans the samples to produce a magnified image for analysis. It is also called SEM microscopy. It gives information about the sample's surface topography and composition. The SEM advanced feature helped to get NP's size. It offers various benefits, as it can analyse sizes of at least 10 nm in transmission mode (T‐SEM), compared to the analysis of nanoparticles on a bulk substrate. This approach to characterising nanocomposites is highly recommendable, valuable, traceable, and accurate. The SEM reveals information about the structure, chemical composition, size, and surface morphology during electron‐sample interaction. EDX analysed the composition of nanoparticles (energy dispersive X‐ray) analysis, and sharp peaks represent nanoparticles.

#### 2.5.6 EDX Spectroscopy

The EDX micro‐analyser in the SEM was used to determine the elemental composition and its concentrations in the specimen. It is a method that depends on the distinctive X‐ray signals that materials subjected to accelerated electrons emit. X‐rays are also produced when secondary electrons (SE) and backscattered electrons (BSE) are formed due to primary incoming electrons interacting with the object. Atoms in the specimen can have their electrons knocked out by high‐energy electrons, which causes the atoms to ionise.

#### 2.5.7 Experimental Design

Ethical approval was obtained from the Institutional Ethical Board of the Institute of Molecular Biology and Biotechnology (IMBB), University of Lahore (2024/IMBB/UOL). Animals were treated according to the Helsinki Declaration and International Guiding Principles for Biomedical Research Involving Animals [24].

Randomly selected 13 Sprague Dawley (SD) rats (7–9 weeks, 130–220 g) were divided into four groups. Rats were housed in cages in a controlled lab setting and fed rodent pellets and tap water. Before the experimentation, the animals were acclimated to the experimental environment for 7 days in the animal house.

#### 2.5.8 Induction of AD

All the SD rats were administered AlCl3 (0.5 mL, 20 mg/kg body weight) for 15 days, except the control group. AlCl_3_‐induced extensive neuronal vacuolation and necrosis of the cerebral cortex and hippocampus.

### 2.6 Animal Grouping and Treatment Design

The rat groups for various treatments have been listed as follows:

Group I: The control group (healthy rats) was left untreated.

Group II: This is an AD‐induced group, containing three subgroups (Table 2) and were treated with Curcumin‐C, Curcumin‐NT, and Curcumin‐T (20 mg/kg/body weight) for 30 days by oral gavage (For detail see Tables 1 and 2).

**TABLE 1:** Nanoformulations and their detail.

Nanoconjugate	Detail
WGX50 targeted (WGX50‐T)	Cys‐capped AuNP‐WGX50‐Insulin
WGX50 non‐targeted (WGX50‐NT)	Cys‐capped AuNP‐WGX50
Curcumin targeted (Curcumin‐T)	Cys‐capped AuNP‐Curcumin‐Insulin
Curcumin non‐targeted (Curcumin‐NT)	Cys‐capped AuNP‐Curcumin
WGX50 base compound	WGX50
Curcumin base compound	Curcumin
Standard drug	Ebixa

Abbreviation: Cys, cysteine.

**TABLE 2:** Experimental Grouping and Treatment Regimen for AD Rat Model.

S. no	Group	Treatment sub‐groups	No. of rats	Dose
I	Control	Untreated	6	0.5 mL
II	Cur‐AuNPs^a^	Curcumin‐C	6	0.5 mL
		Curcumin‐T	6	0.5 mL
		Curcumin‐NT	6	0.5 mL
III	WGX50‐AuNP^a^	WGX50‐C	6	0.5 mL
		WGX50‐T	6	0.5 mL
		WGX50‐NT	6	0.5 mL
IV	Standard	Ebixa	6	0.5 mL

^a^ Cur‐AuNP, curcumin gold nanoparticle.

Group III: This group also comprised three subgroups subsequently treated with WGX50, WGX50‐NT, WGX50‐T (20 mg/kg/body weight) for 30 days by oral gavage.

Group IV: This group was treated with the standard drug Ebixa (20 mg/kg/body weight) for 30 days by oral gavage.

After 24 h of the oral administration of the last doses of all nanoconjugates and standard drugs, the rats were subjected to multiple behavioural studies. Subsequently, they were slaughtered by euthanasia. Blood and brain tissues (cortex and hippocampus) were subjected to further studies.

### 2.7 Organ Collection

After 24 h from the last dose, the rats were dissected to collect their brains. The brain was collected in a falcon tube filled with 10% formalin. These tubes were sent for examination to the histopathology laboratory, The University of Lahore.

### 2.8 miRNA Analysis

#### 2.8.1 Blood Collection and Serum Isolation

Blood samples were collected from all the SD rats using EDTA‐coated tubes. After collection, the tubes were gently inverted several times to ensure proper mixing of the blood with the EDTA. The samples were then centrifuged at 3000 rpm for 10 min at 4°C to separate the plasma from the blood cells. The isolated plasma was stored at −80°C for long‐term preservation for analysis as required.

#### 2.8.2 RNA Extraction

Total RNA, including miRNA, was extracted from the plasma of treated rats using a miRNA extraction kit according to the manufacturer instruction. The quality and quantity of the extracted RNA were checked using a Nanodrop. Briefly:

A solution of lysis buffer was introduced to Eppendorf tubes and vortexed for 10 min at room temperature, followed by incubation for 30 min. 20 μL of 2 M sodium acetate solution (pH 5.2) was added post‐incubation to each Eppendorf tube followed by addition of 180 μL water‐saturated phenol and 40 μL chloroform for 2 min with vigorous vortexing. All the tubes were centrifuged at 6000 rpm for 10 min. The supernatant was transferred to a new 1.5 mL centrifuge tube, and 35% absolute ethanol was added to the upper phase volume (108 μL for 200 μL of upper phase), followed by extensive mixing. Centrifugation at 12,000 rpm for 30 s resulted in large RNA molecules binding to the column membrane. The filtrate was washed with 70% absolute ethanol. The mixture was transferred to an RNA column in a new collection tube and incubated for 1 min, followed by the addition of 200 μL of wash buffer 2. The mixture was then allowed to stand for an additional 1 min. The tubes were centrifuged at 12,000 rpm for 1 min.

The RNA column was removed from the wash buffer 2 solution and placed into a new 1.5 mL microcentrifuge tube. A pre‐heated (65°C) release buffer was poured into the column for RNA elution. For miRNA purification, the tubes were incubated for 3 min, followed by centrifugation at 12,000 rpm for 3 min. The end product was dissolved in TE buffer at pH 8.0.

#### 2.8.3 cDNA Synthesis

Total miRNA was reverse transcribed into cDNA using a reverse transcription kit following the manufacturer's protocol. Reaction 1: About 8 μL RNA and 2 μL 5× gDNA Buffers (Total volume = 10 μL) in PCR tubes. The mixture was centrifuged for 5 s, followed by incubation at 42°C for 3 min. Reaction 2: About 2 μL 10xking RTmix, 1 μL RT enzyme mixture, 2 μL primer mix and 5 μL RNase‐free H_2_O (Volume = 10 μL). Reactions 1 and 2 were mixed and incubated at 42°C for 15 min and at 95°C for 3 min. The synthesised cDNA was placed into ice.

#### 2.8.4 Relative Quantitative Analysis

Real‐time quantitative PCR was used to quantify APP and miR‐146a‐5p. Using the delta–delta Ct formula, the relative expression level of each microRNA was determined as a normalised fold change: 2^−(ΔΔCt)^.

#### 2.8.5 Histopathology

The blood and brain tissues (cortex and hippocampus) of treated rats (Table 2) were subjected to histopathological examination.

### 2.9 Statistical Analysis

The Mann–Whitney *U* test and one‐way analysis of variance (ANOVA) were used to analyse the data. *p*‐values ≤ 0.05 were considered statistically significant. All of the statistical tests were carried out using SPSS and GraphPad Prism.

## 3 Results

The study prepared cysteine‐capped nanoparticle as an effective linker molecule for the functionalization of AuNP with therapeutic agents, WGX50 and curcumin. The thiol (–SH) group of cysteine forms a stable Au–S bond with AuNP surface to prevent drug desorption under physiological conditions. The amine (–NH_2_) and carboxyl (–COOH) groups provide versatile reactive sites for covalent attachment of drug molecules, maintaining their bioactivity.

The absorbance spectra showed distinct interaction patterns where cysteine‐capped AuNPs demonstrated a strong SPR band around 3000 cm^−1^ and more pronounced peaks in the 1500–2000 cm^−1^ and < 1000 cm^−1^ regions.

### 3.1 UV–Vis Spectrophotometry

**FIGURE 1:**
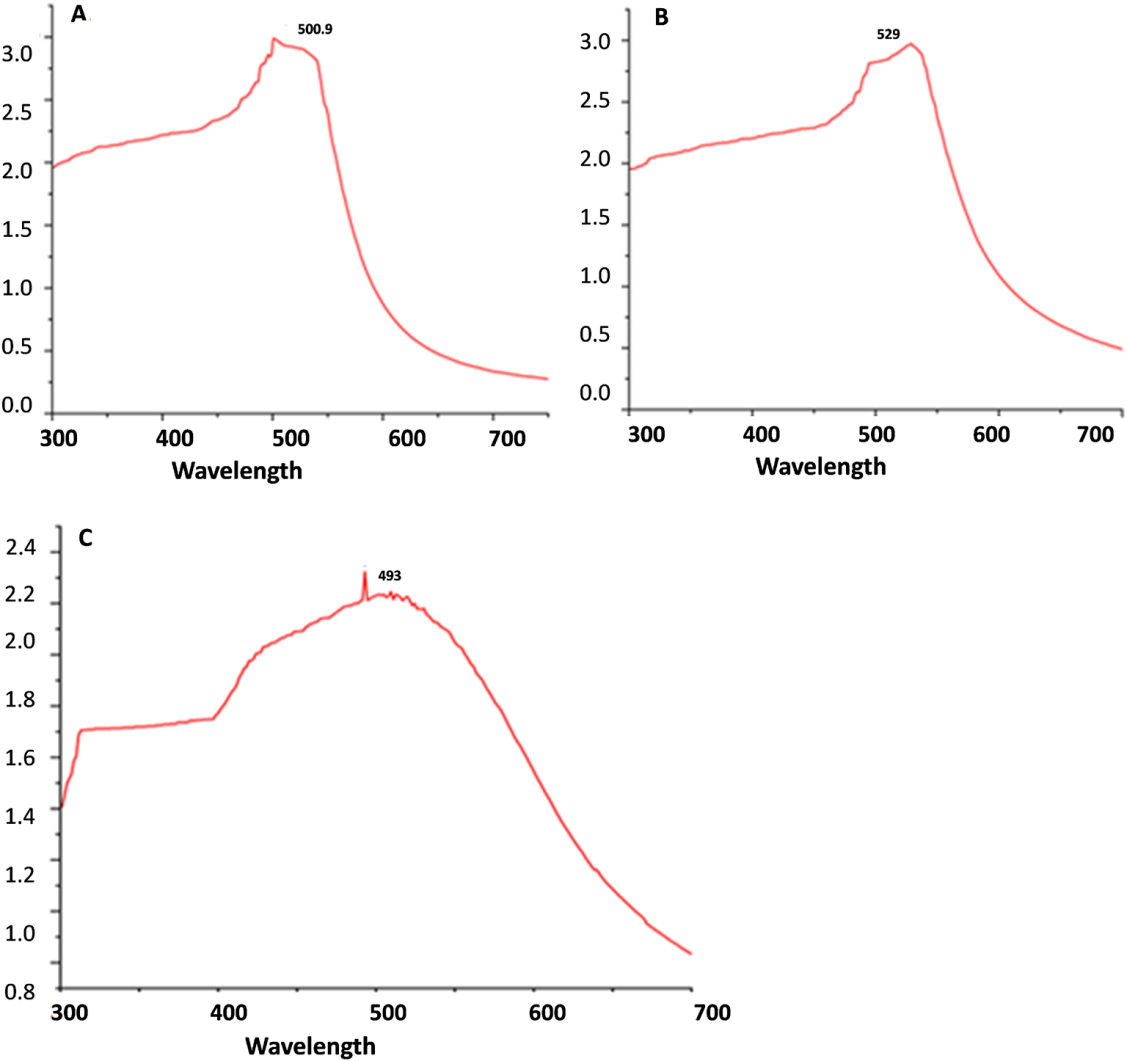
UV–Vis absorbance spectra of (A) Pure‐AuNPs (B) Cys‐AuNPs (C) Cur‐AuNPs (Curcumin‐NT).

UV spectroscopy is a valuable tool for characterising NPs during the formulation process. The colours of the AuNPs produced vary from red to purple. The purple colour indicates larger particles, and red indicates smaller AuNPs. To better understand the size and distribution, we need to analyse the UV–Vis spectroscopy data. UV–Vis test was performed for nine samples. A peak at 500.5 nm was observed for the AuNP prepared by sodium tri‐citrate in graph A (Figure 1), the peak of AuNPs coated with cysteine at 529 nm in graph B, and the AuNPs synthesised using curcumin for the reduction of the gold show a peak at 493 nm in graphs C, respectively (Figure 1). Considering errors, the maximum absorbance of AuNPs was at 500, 529, and 493 nm (Figure 1A–C).

### 3.2 UV–Vis Curcumin, WGX50

**FIGURE 2:**
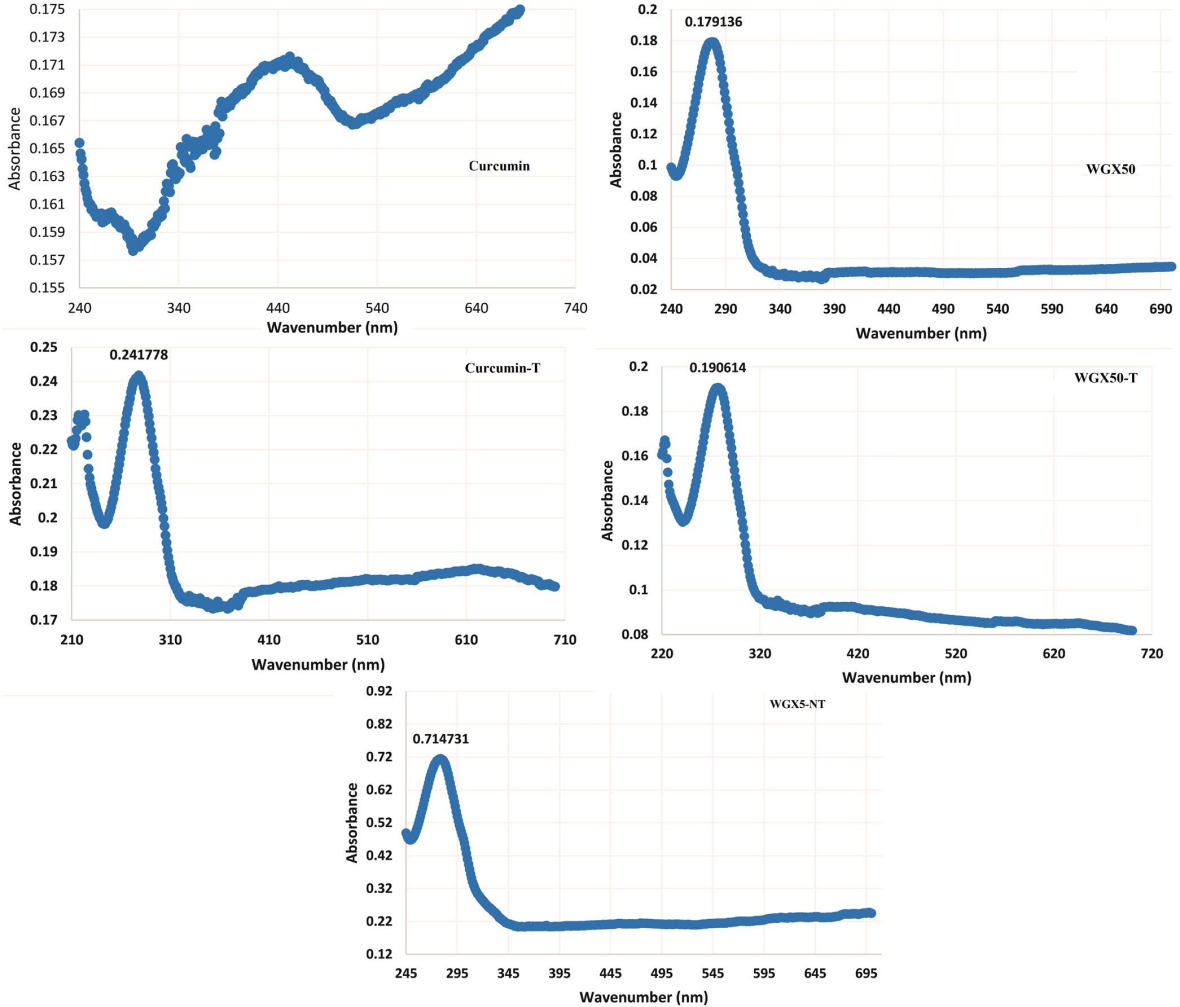
UV–vis Curcumin, WGX50, Curcumin‐T, WGX50‐T, and WGX50‐NT.

The UV–Vis absorbance spectra reveal differences between curcumin, WGX50, and their targeted formulations. The curcumin and its targeted form show characteristic absorbance peaks around 300–350 nm, with Curcumin‐T exhibiting a higher peak intensity (0.241778), indicating possible structural modifications or enhanced solubility. The WGX50 and WGX50‐T display strong absorbance peaks around 290–320 nm, with WGX50‐T showing slightly increased absorbance (0.190614), suggesting successful functionalization (Figure 2). In contrast, WGX50‐NT has a significantly higher peak absorbance (0.714731), possibly due to aggregation or different surface interactions. The shift in absorbance intensities and peak positions across samples indicates modifications in optical properties, which may be attributed to molecular interactions, structural modifications, or encapsulation efficiency.

### 3.3 Fourier Transform Infrared Spectroscopy (FTIR) Analysis

**FIGURE 3:**
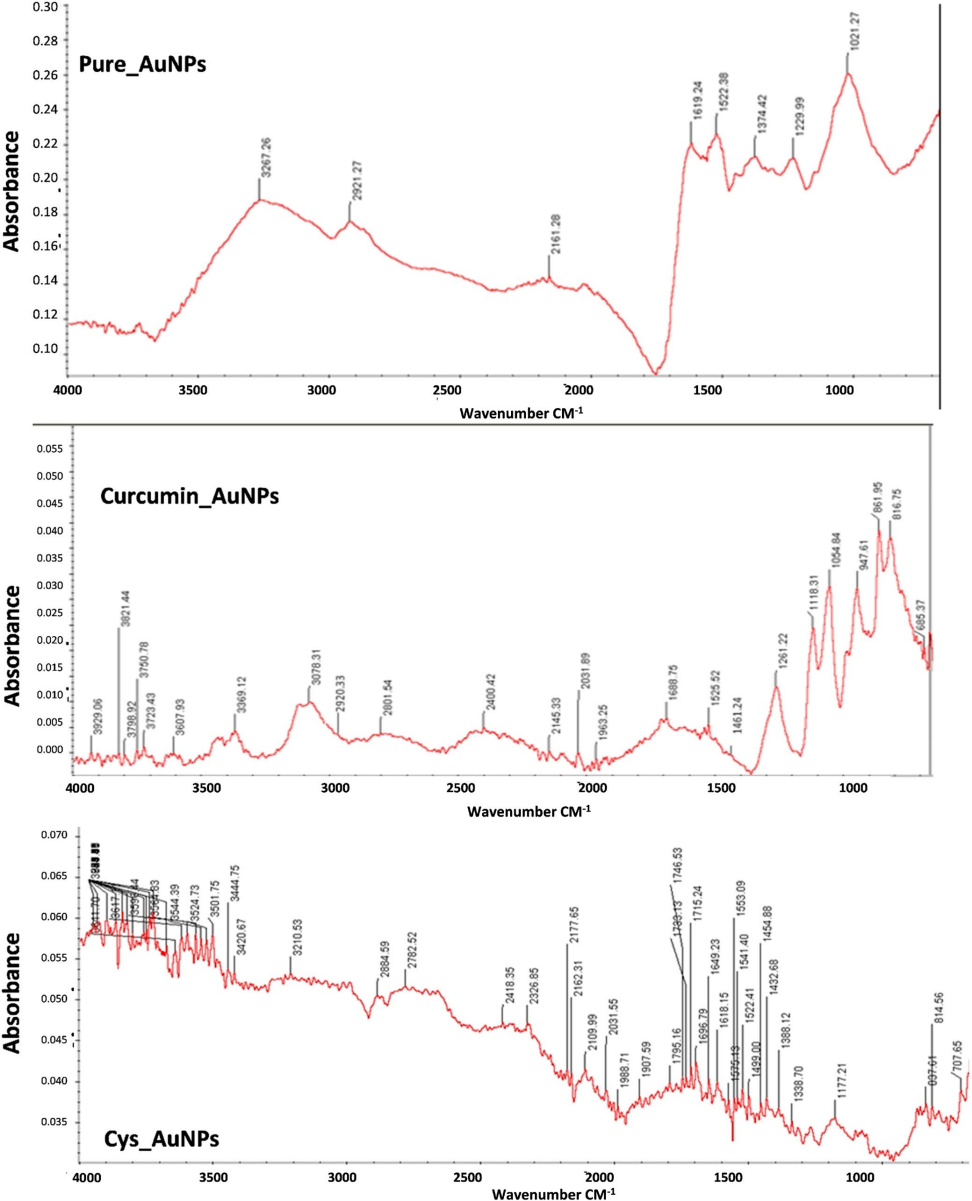
FTIR of pure‐AuNPs, curcumin‐AuNPs, and Cys‐AuNPs.

Pure AuNPs: The absorbance spectrum of pure AuNPs exhibits a broad peak around 3000 cm^−1^, which is characteristic of the surface plasmon resonance (SPR) of AuNPs (Figure 3). The presence of other smaller peaks in the range of 1000–1500 cm^−1^ may be due to various vibrational modes of the nanoparticles.

Curcumin_AuNPs: The absorbance spectrum of curcumin‐capped AuNPs shows a strong peak around 3000 cm^−1^, similar to pure AuNPs, indicating the presence of the SPR band. However, there are some additional peaks in the range of 1500–2000 cm^−1^ and below 1000 cm^−1^, which may be due to the vibrational modes of the curcumin molecules adsorbed on the surface of the AuNPs. These curcumin peaks have a significant influence on the vibrational properties of the AuNPs.

**FIGURE 4:**
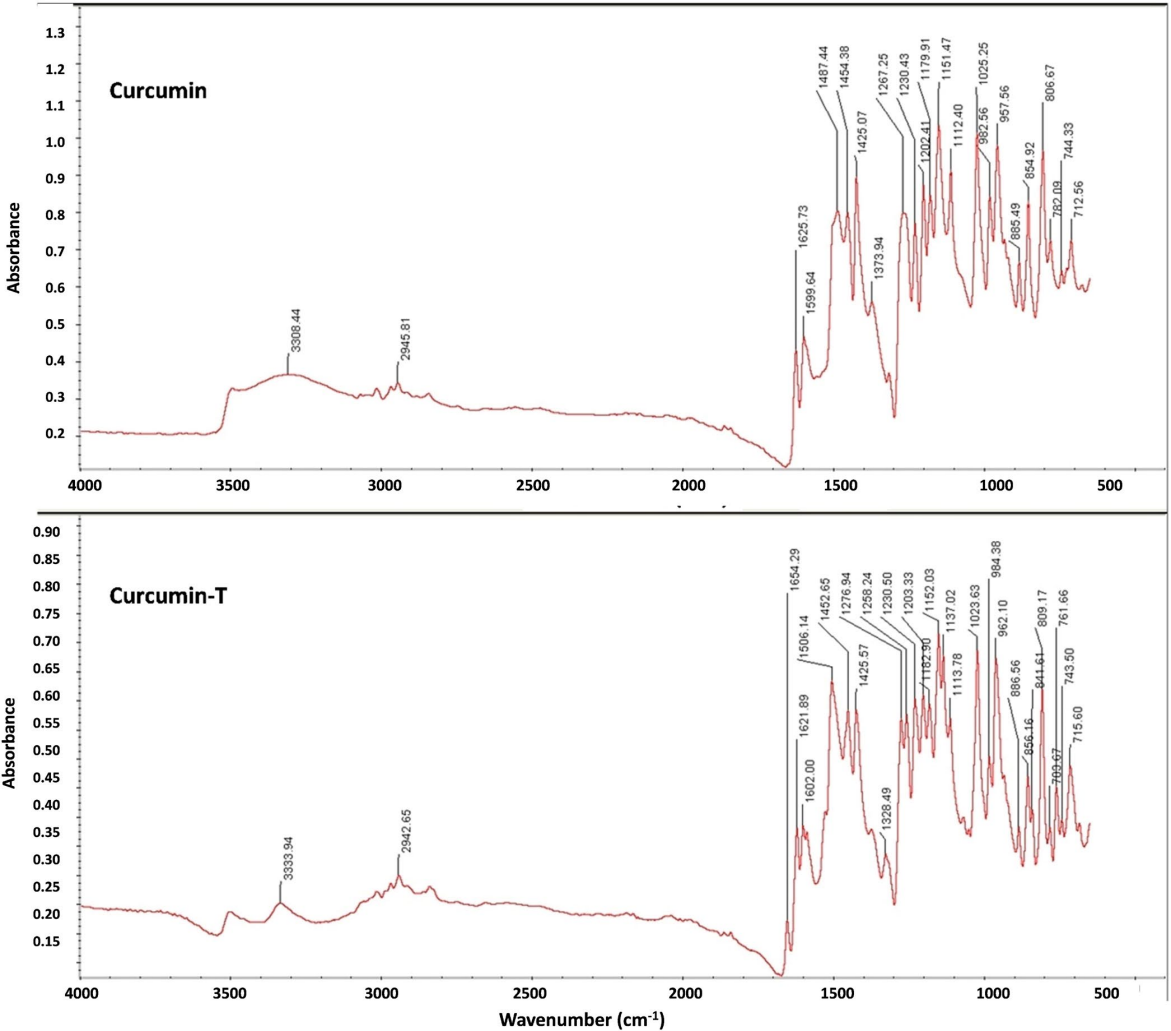
FTIR of curcumin and curcumin‐T (curcumin‐AuNPs‐insulin). The upper panel displays the spectrum of pure Curcumin, with absorption peaks at 3398.44 cm^−1^ (wide O‐H stretching) and 2944.81 cm^−1^ (C‐H stretching). The lower panel, with absorption peaks at 3333.94 cm^−1^ (O‐H stretching) and 2942.66 cm^−1^ (C‐H stretching) (Curcumin‐T), shows the presence of Curcumin in the nanocomposite. Additional peaks in the Curcumin‐T suggest interactions between Curcumin and the AuNPs‐insulin.

Cys_AuNPs: The absorbance spectrum of cysteine‐capped AuNPs (Figure 3) exhibits a strong SPR band around 3000 cm^−1^, similar to the other two types of AuNPs. The peaks in the range of 1500–2000 cm^−1^ and below 1000 cm^−1^ are more obvious compared to curcumin‐capped AuNPs. This shows that cysteine has a more significant effect due to interactions between cysteine and the AuNP surface (Figure 4).

The absorbance spectrum of pure curcumin displays an obvious peak around 3400 cm^−1^, which is associated with the O‐H stretching of phenolic hydroxyl groups in curcumin. The peak at approximately 2900 cm^−1^ is also obvious which corresponds to the C‐H stretching. There are several peaks in the 1500–1800 cm^−1^ range, which are characteristic of the C=O and C=C stretching in the curcumin molecule. The presence of multiple peaks below 1500 cm^−1^ indicates various vibrational modes of the molecule. The absorbance spectrum of curcumin‐T exhibits a similar peak around 3400 cm^−1^, showing the O‐H stretching vibrations of the phenolic hydroxyl groups. The peak at 2900 cm^−1^ is also present; however, the peaks in the range of 1500–1800 cm^−1^ are less pronounced, which could be due to the interaction between curcumin and the AuNPs or the insulin‐targeting moiety. The peaks below 1500 cm^−1^ are also present, but may indicate an altered molecular environment of curcumin when conjugated with AuNPs and the targeting agent.

### 3.4 FTIR Analysis WGX50, WGX50‐T and WGX50‐NT

**FIGURE 5:**
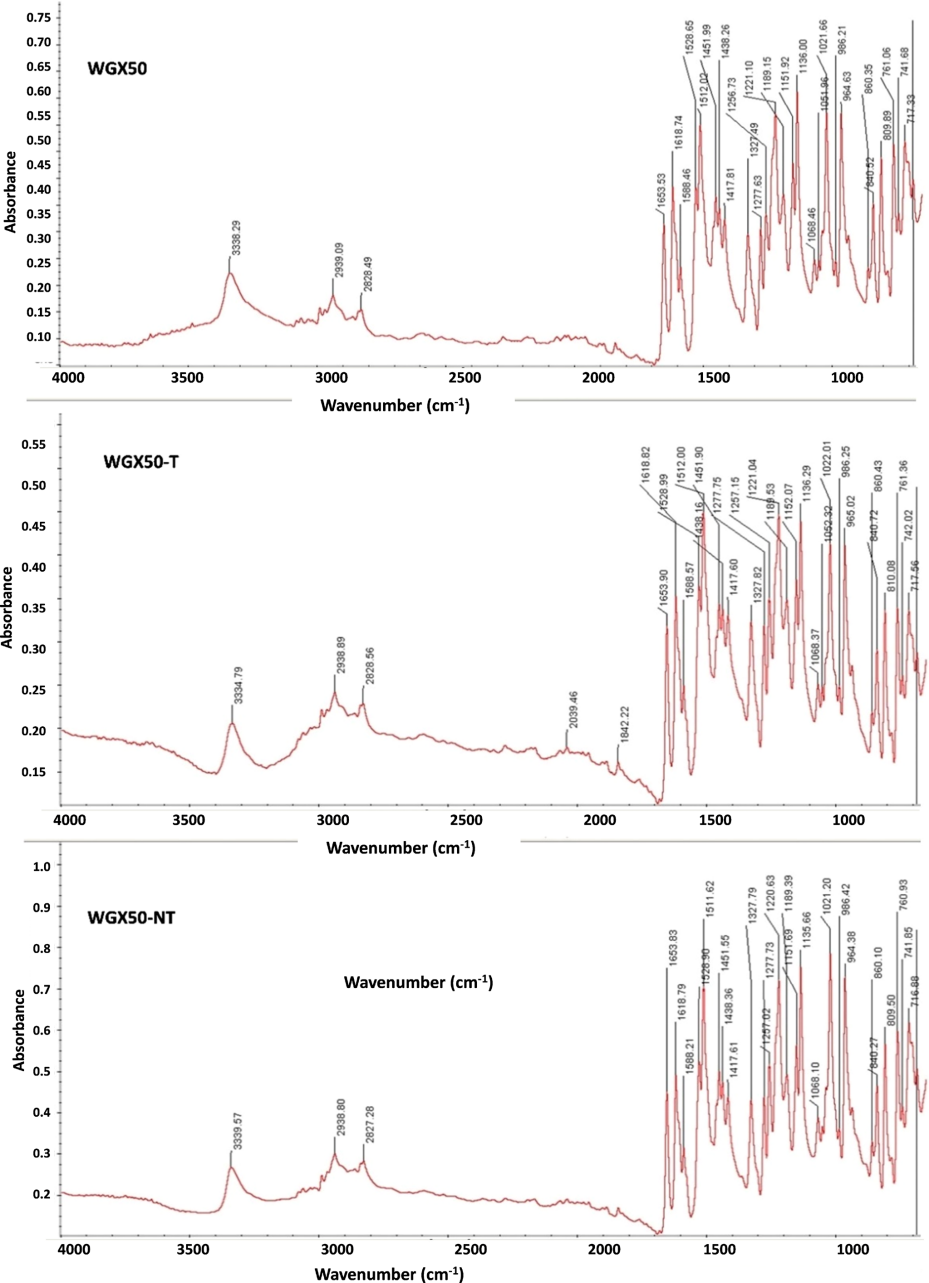
FTIR of WGX50, WGX5‐T (AuNPs‐WGX50‐insulin), WGX50‐NT (AuNPs‐WGX50). The top panel: Spectrum of WGX50, absorption peaks 3388.29 cm^−1^. The middle panel shows the WGX50‐T (peaks at 3384.79 cm^−1^ and 2928.96 cm^−1^). Additional peaks suggest interactions. The bottom panel: WGX50‐NT, with absorption peaks at 3325.57 cm^−1^ and 2908.80 cm^−1^. Peaks in all three spectra from 1500 cm^−1^ to 500 cm^−1^ correspond to various C‐O, C‐C, and other functional group vibrations, providing bonding within each nanocomposite.

The FTIR spectrum of WGX50 exhibits a broad peak around 3300–3400 cm^−1^, which is typically associated with the O‐H stretching vibrations of alcohols or phenols (Figure 5). There is also a peak around 2900 cm^−1^ and several other peaks in the range of 1500–1800 cm^−1^, which could be due to the C‐H, C=O, and C=C stretching vibrations, respectively. Multiple peaks below 1500 cm^−1^ indicate various bending and twisting vibrational modes.

The middle FTIR spectrum in Figure 5 of WGX50‐T (WGX50 conjugated with AuNPs and insulin). The spectrum is similar to that of WGX50, with a broad peak (3300–3400 cm^−1^) and also a peak around 2900 cm^−1^. The peaks in the range of 1500–1800 cm^−1^ show slight shifts or changes in intensity, which could be due to the interaction between the WGX50 and the AuNPs or insulin. The peaks below 1500 cm^−1^ could indicate alterations in the molecular environment due to conjugation (See Figure 5).

The bottom graph of the FTIR spectrum of WGX50‐NT (Figure 5), in which WGX50 is conjugated with AuNPs without insulin, is very similar to that of WGX50 and WGX50‐T, with the same broad peak around 3300–3400 cm^−1^ and 2900 cm^−1^. The peaks in the range of 1500–1800 cm^−1^ and below 1500 cm^−1^ are also present, with slight shifts in intensity similar to those observed in WGX50‐T.

The main differences in FTIR of WGX50, WGX5‐T (AuNPs‐WGX50‐insulin), WGX50‐NT (AuNPs‐WGX50) spectra may involve slight shifts in peak positions, particularly in the range of 1500–1800 cm^−1^ and below 1500 cm^−1^. These differences may be due to the interactions between WGX50 and the AuNPs, or due to the presence or absence of insulin. The conjugation with AuNPs and insulin may alter the molecular environment of WGX50. However, the overall spectral patterns remain constant, indicating that the key functional groups of WGX50 are preserved in both WGX50‐T and WGX50‐NT.

### 3.5 DLS Analysis

**FIGURE 6:**
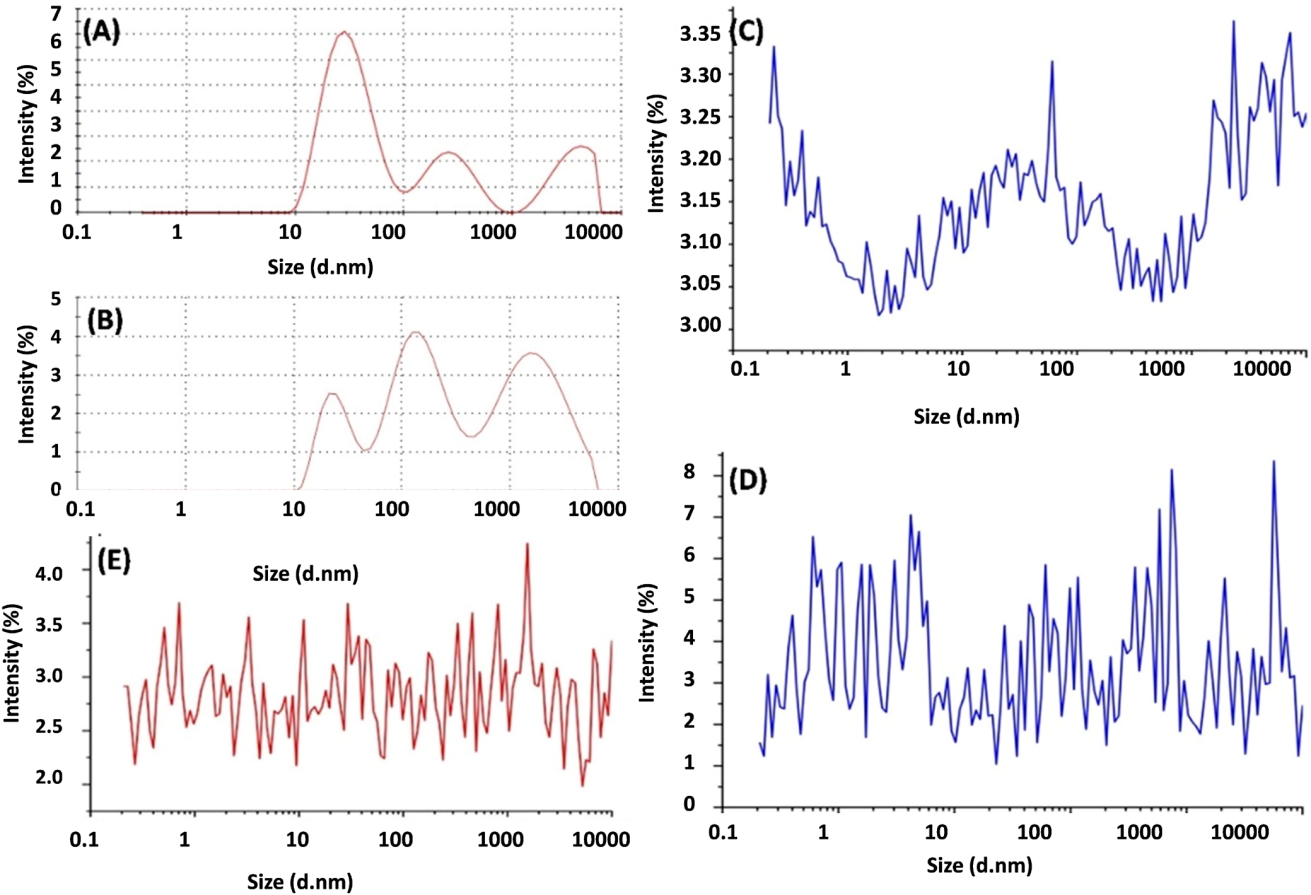
DLS intensity size distribution graphs for different AuNP formulations. (A) size distribution of Pure‐AuNPs with a peak around 100 nm. (B) size distribution of Cys‐AuNPs, which also peaks around 100 nm. (C) size distribution for curcumin‐NT, displaying a broader size distribution, suggesting aggregation. (D) size distribution for Curcumin‐T. (E) size distribution of WGX50‐NT.

The DLS analysis indicates differences in size distribution and intensity among pure‐AuNPs, Cys‐AuNPs, Curcumin‐NT, Curcumin‐AuNPs, and Curcumin‐T, indicating variations in particle size, concentration, and possibly aggregation states due to the different surface modifications and interactions (Figure 6A–E). Pure AuNPs exhibit a polydisperse nature, displaying a broad size distribution with peaks at approximately 10 nm and 100 nm. Cys‐AuNPs, exhibiting a broad size distribution (around 10 nm and 100 nm) (Figure 6B), are similar to pure‐AuNPs. However, the intensity is lower compared to pure‐AuNPs, which might indicate a change in the scattering properties due to the cysteine capping. Graphs (Figure 6C,D) depict curcumin complexes with and without insulin, respectively. Both graphs show a more complex and irregular pattern, with multiple peaks across a wide range of sizes. The intensity in graph (Figure 6C) is generally lower than in graph (Figure 6D), suggesting that the addition of insulin might affect the size distribution. The irregular pattern in both graphs could be due to the interaction between curcumin, AuNPs, and the nanotube structure.

### 3.6 XRD

**FIGURE 7:**
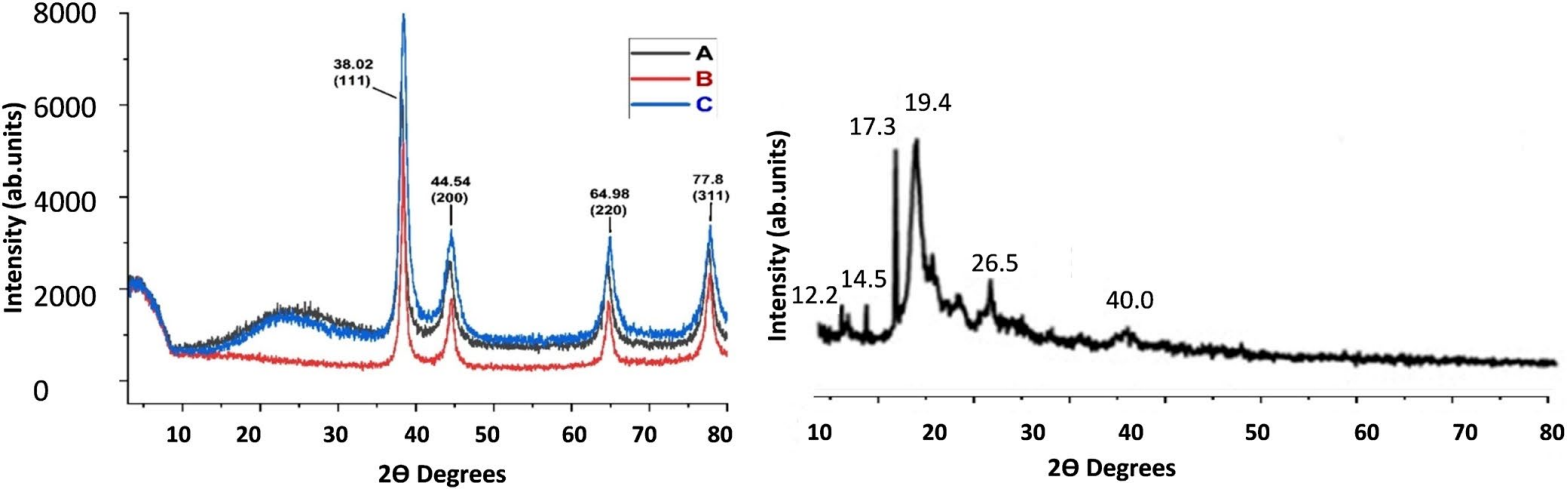
XRD patterns of AuNP formulations: (A) Pure‐AuNPs, (B) Cys‐AuNPs, and (C) Curcumin‐NT. The diffraction peaks are labelled with the Miller indices corresponding to the face‐centered cubic (FCC) structure of gold. Peaks at 2θ angles of 38.02° (111), 44.54° (200), 64.98° (220), and 77.8° (311).

The XRD peaks for AuNPs indicate the formation of distinct, well‐defined phases. The XRD diffractogram shows the diffraction peaks at 2θ = 38.12°, 44.32°, 64.54°, and 77.72° corresponding to the crystallographic planes of (111), (200), (220), and (311), respectively (Figure 7a–c). Crystalline AuNPs have been represented with four peaks corresponding to the standard Bragg reflections (111), (200), (220), and (311) of a face‐centered cubic lattice. The intense peak at 38.12 represents preferential growth in the (111) direction. The characteristic peaks of the Au + signal at 2θ = 38.12° confirm the presence of Au + in the AuNPs nanocomposite. The peaks at 2θ = 38.12°, 44.32°, and 64.54° are assigned to the (111), (200), and (220) planes of metallic gold (Figure 7). The appearance of discrete phases for AuNPs indicates the synthesis of crystalline NPs. The peaks represent the purity of NPs.

**FIGURE 8:**
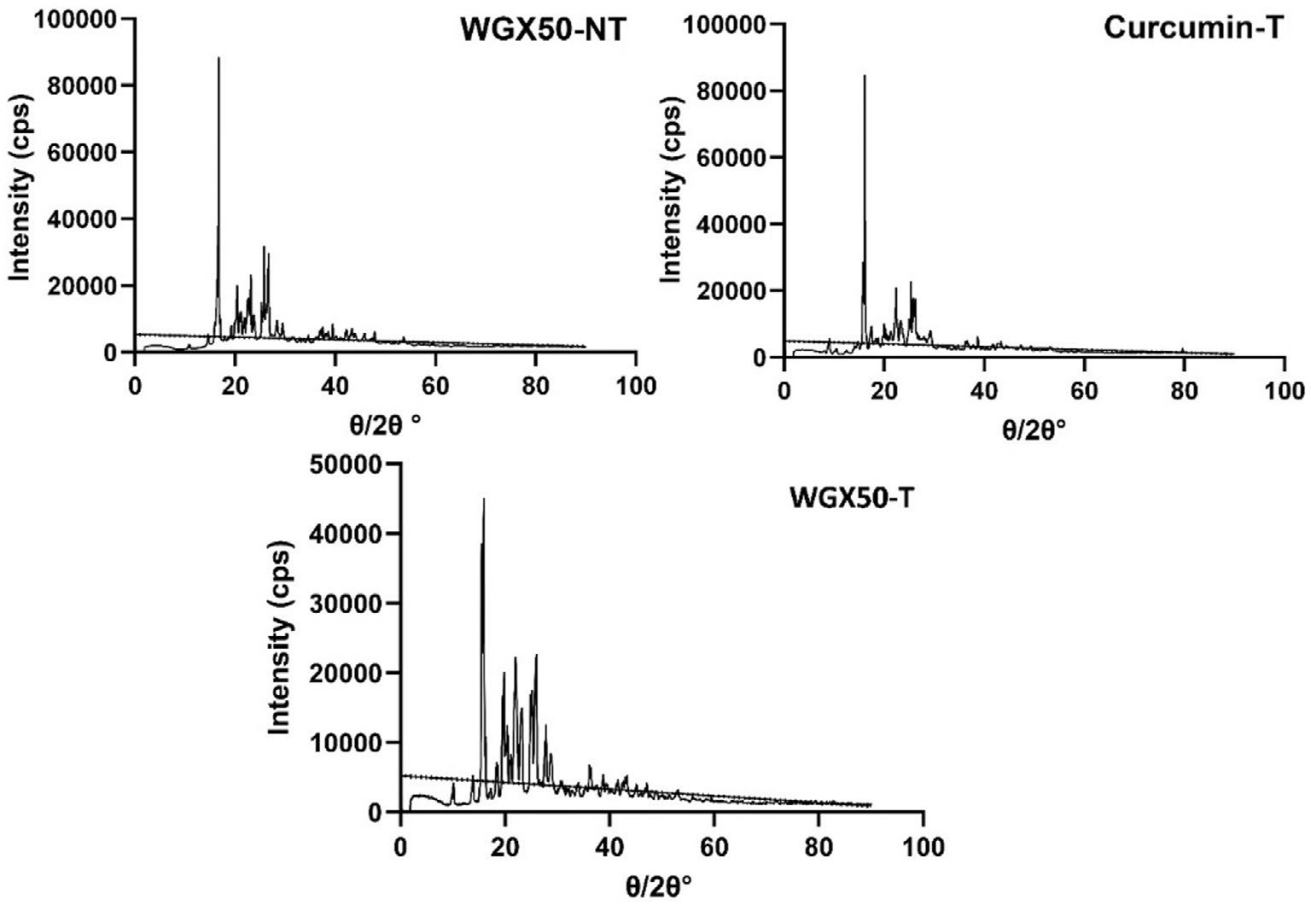
The XRD patterns for WGX50‐NT, WGX50‐T, and Curcumin‐T. Each panel shows the diffraction intensity as a function of the scattering angle (2θ). The WGX50‐NT pattern (top left) shows multiple peaks indicative of a crystalline structure. The WGX50‐T pattern (top right). The Curcumin‐T (bottom) shows a complex diffraction pattern.

The XRD patterns of WGX50‐NT, WGX50‐T, and Curcumin‐T exhibit distinct peaks, indicating varying degrees of crystallinity. All three spectra display sharp diffraction peaks, suggesting a crystalline nature (Figure 8). The highest peak intensities have been observed in the WGX50‐NT and Curcumin‐T, suggesting a well‐ordered structure. The WGX50‐T demonstrated lower peak intensity, indicating structural modifications resulting from interaction with targeting agents. The presence of multiple sharp peaks at lower 2θ values suggests the existence of multiple crystalline phases.

### 3.7 SEM

**FIGURE 9:**
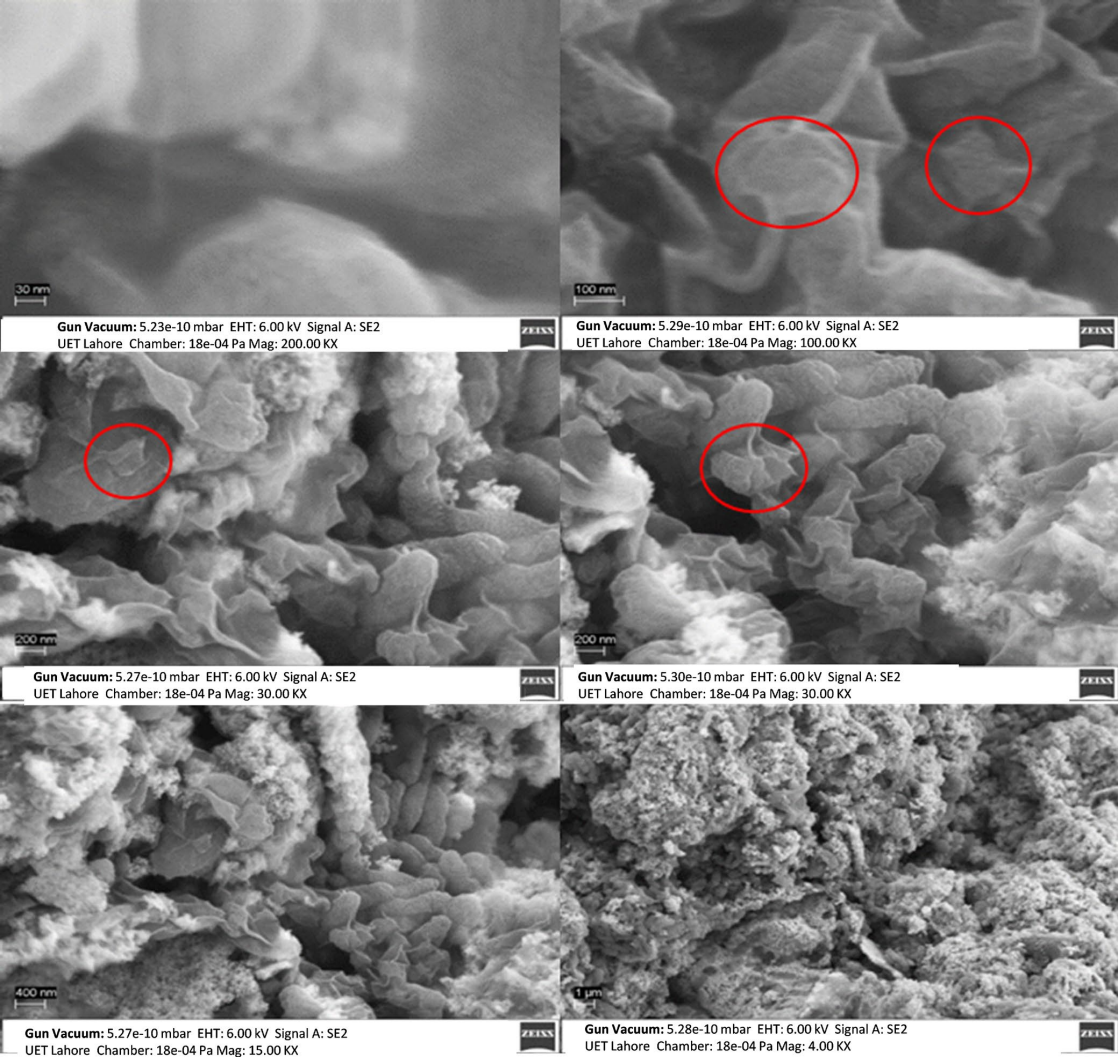
SEM images of AuNPs at 1 μm, 400 nm, 200 nm, 100 nm, and 30 nm. At the 1 μm scale, the AuNPs appear as an aggregate of smaller particles. As the magnification increases to 400 nm and 200 nm, nanoparticles become more distinguishable, showing a range of shapes and sizes. At the 100 nm and 30 nm scales, the detailed surface texture and aggregation patterns of the AuNPs are evident. The red circles highlight regions of interest where the AuNPs features are obvious.

**FIGURE 10:**
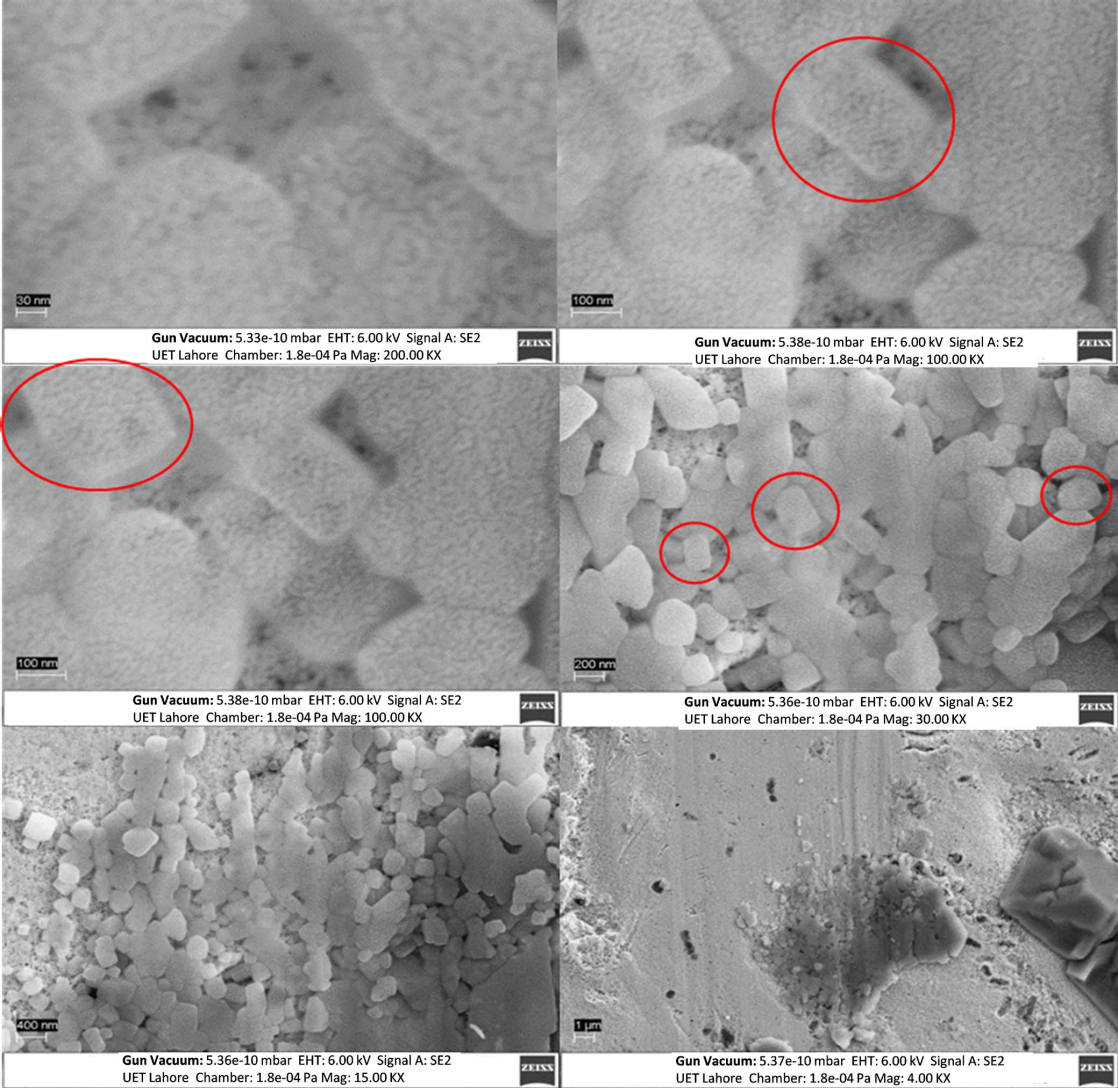
SEM images of Cys‐AuNPs at 30 nm, 100 nm, 200 nm, 400 nm, and 1 μm. At the 30 nm scale, individual nanoparticles are visible and showing shape and size. 100 nm and 200 nm offer a clearer view of the particles' surface and aggregation. The 400 nm and 1 μm scales reveal a more comprehensive view of the Cys‐AuNPs and larger aggregates. The red circles Cys‐AuNPs particle size and shape.

**FIGURE 11:**
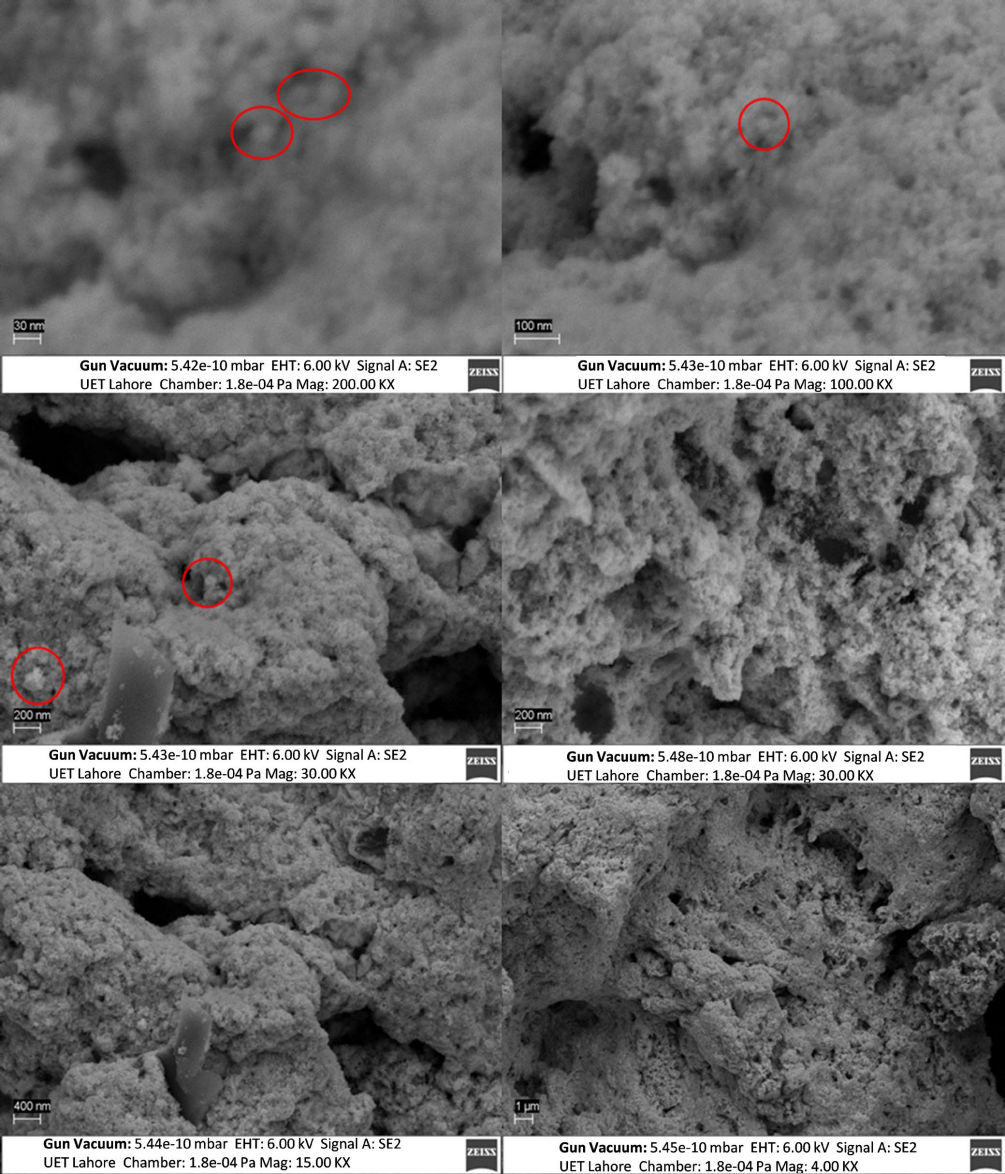
SEM images showing morphology of Curcumin‐T at magnifications of 30 nm, 100 nm, 200 nm, 400 nm, and 1 μm, illustrating the surface topography and particle aggregation at different scales.

Figures 9, 10, 11 illustrate the synthesised s structural features of the nanocomposite and elemental composition using SEM equipped with EDX. It shows the surface morphology of nanocomposites at different magnifications of 30 nm, 100 nm, 200 nm, 400 nm, and 1 μm. This magnified image shows the irregular spherical shapes of AuNPs with a diameter of approximately 30–40 nm (Figure 9). The SEM reveals information about the solid specimen when the high‐energy electron beam makes an array of signals focused on the targeted sample. The cysteine‐capped AuNPs viewed under SEM show irregular cubic morphology with a diameter of approximately 35–40 nm (Figure 10). The chemical AuNPs have shown aggregated particles of varying sizes; the approximate diameter of the particles was less than 10 nm. The Ins‐cur‐capped AuNPs (Curcumin‐T) show irregular cubic morphology with a diameter of approximately 100 and 450 nm (Figure 11).

### 3.8 SEM Analysis of Curcumin‐T

The SEM images display the morphological features of Curcumin‐T. In the Curcumin‐T images (50,000× and 100,000×), the surface exhibits a rough and aggregated structure. The higher magnification image reveals intricate surface patterns, indicating the presence of possible curcumin‐loaded nanostructures or modifications. The WGX50‐NT sample shows a relatively smooth and uniform surface with smaller dispersed particles, suggesting minimal surface modification. This smooth morphology may indicate weaker interaction or lower efficiency in encapsulation or targeting applications. In contrast, the WGX50‐T sample (at 100,000×) exhibits an aggregated structure, similar to Curcumin‐T, but with slightly smoother features. This suggests a modification in surface morphology due to targeting, which may potentially improve material interaction.

### 3.9 SEM Analysis of WGX50‐T, and WGX50‐NT

**FIGURE 12:**
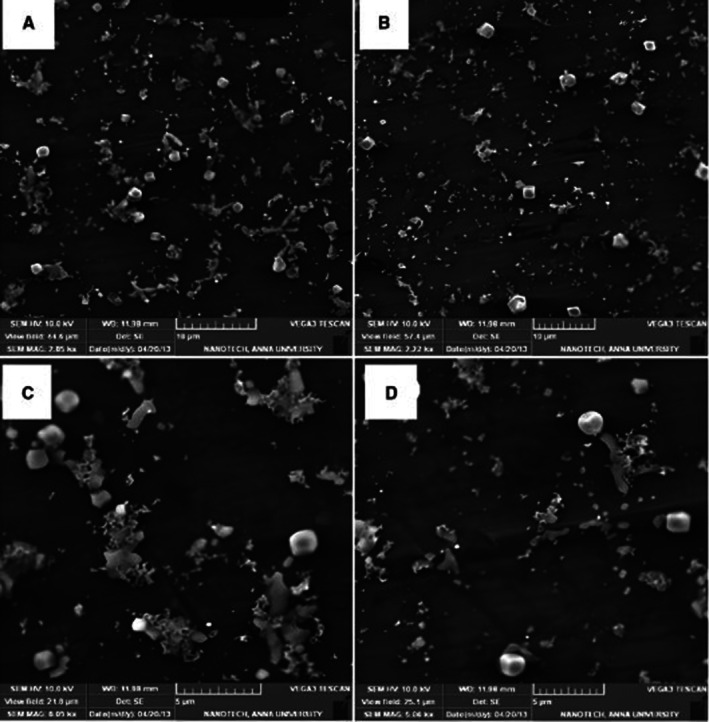
SEM images of comparing the surface morphology. (A, C) WGX50‐NT and (B, D) WGX50‐T. (A, C) shows WGX50‐NT with a relatively uniform distribution. (B, D) WGX50‐T nanoparticles appear more dispersed with fewer aggregates.

**FIGURE 13:**
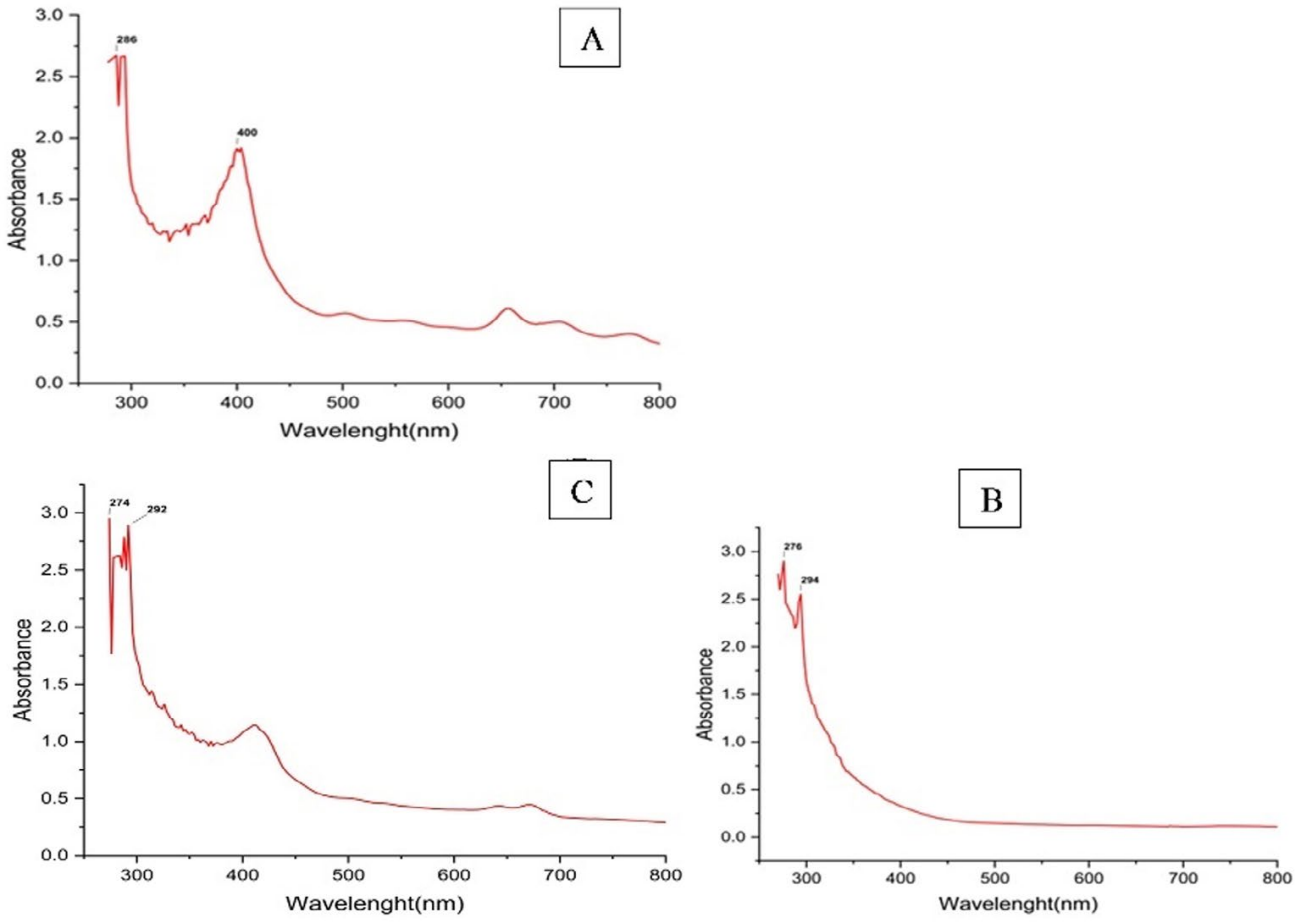
SEM images comparing the surface morphology. (A) WGX50‐NT and (B) WGX50‐T. (A) shows WGX50‐NT with a relatively uniform distribution. (B) WGX50‐T nanoparticles appear more dispersed with fewer aggregates.

The SEM depicts the surface morphology of WGX50‐NT and WGX50‐T (Figure 12A–D), each taken at different magnifications, suggesting a variety of particle sizes and shapes, with some particles appearing to be clustered together. Figure 13B provides a distribution of particles that appears more uniform. The particles in image (B) appear to be smaller and more dispersed across the surface.

### 3.10 EDX

The EDX analysis was performed on the synthesised AuNPs to determine their elemental composition. The EDX confirms the presence of Au and other elements, as shown in Tables S1 and S2. The gold carries 13.46% of the total weight % of the pure AuNPs formed; other elements present in the AuNPs with a more significant weight % are oxygen, nitrogen, and carbon (Table S1). The total weight % of gold present in cysteine‐capped AuNPs is 52.13%, while the elemental presence of carbon, oxygen, and sodium is 12.88%, 14.46%, and 14.29% (Table S2).

### 3.11 UV–Vis Spectrophotometry

UV–Vis test was performed for nine samples. A peak at 300 nm and 400 nm was observed for the WGX50 (Figure 8), the peak of AuNPs coated with gx50 at 300 nm and 429 nm in graph B, and the AuNPs synthesised using insulin‐capped gx50‐AuNPs show a peak at 375 nm and 493 nm in graphs C, respectively (Figure 13A–C).

### 3.12 Histopathology

Rats from both the control and treatment groups were humanely euthanized for subsequent analysis, and the cortex and hippocampus were separated, cleaned with cold phosphate‐buffered saline, and then fixed in 10% formalin. For both tissues, paraffin‐embedded blocks were made. Haematoxylin and eosin were used to stain the paraffin sections after sectioning, and they were then examined under a light microscope.

The aluminium chloride (AlCl3) was procured from Merck Life Sciences Private Limited. All rats were administered with AlCl_3_ 0.5 mL (100 mg/kg body weight) for 15 days to induce toxicity in the brain, which causes aggregation of amyloid and neurofibrillary tangles, abbreviated as (a) and (b). Abixia is a new herbal remedy derived from the *A. indica* plant and has been studied for its potential benefits in treating AD. Preclinical research suggests that by reducing the production of amyloid‐β (Aβ) plaques and enhancing cognitive function, abixia may help alleviate the symptoms associated with AD. As a standard drug, we used it in rats to treat AD.

**FIGURE 14:**
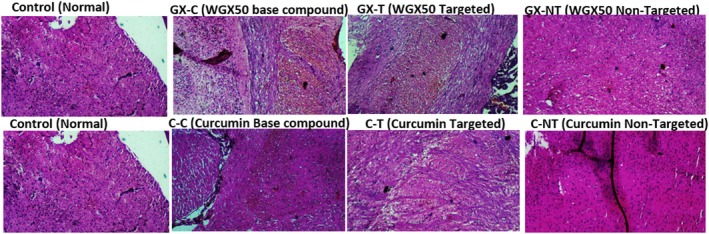
Brain histological analysis of control groups and nanoconjugates treated. Histopathological analysis of AD rat models treated with WGX50 and Curcumin‐based nanoformulations. Representative stained sections (5 μm thickness; scale bar = 50 μm). Treatment with WGX50 and Curcumin nanoformulations (T and NT groups) demonstrates neuroprotective effects.

We used immunohistochemistry to assess the levels of Aβ plaques in the brains of AlCl3‐induced SD rats. A shown in Figure 14, the WGX50‐NT and WGX50‐T had a direct impact on Aβ oligomers and it could cross the BBB with insulin to enhance cognition. Additionally, WGX50‐NT and WGX50‐T therapy drastically reduced the buildup of Aβ oligomers in the cerebral cortex. These findings suggest that WGX50‐T is a candidate conjugate for the treatment of AD. The administration of WGX50 and WGX50‐T drastically reduced Aβ plaque deposition or polymerisation by approximately 80%–90% and protected neurons from damage. Overall, the comparative analysis showed that nanoformulations may influence therapeutic performance in AD models, with targeted delivery (Curcumin‐T and WGX50‐T) demonstrating the potential for reversing neurodegeneration (File S4). These findings highlighted the scientific value of enhanced drug delivery systems for improving brain bioavailability and offering neuroprotective outcomes compared to non‐targeted and conventional treatments.

Quantitative histopathological analysis of brain sections using ImageJ revealed differences in neuroprotective efficacy of the nanoformulations (Figure 14, Table 3). The control group exhibited a mean intensity of 125.93, representing normal neuronal morphology. The WGX50‐T group showed reduced intensity (108.36), an indication of improved neuronal preservation and reduced neurodegeneration. The WGX50 and WGX50‐NT displayed higher mean intensities (≈122). The Curcumin group demonstrated the lowest mean intensity (92.42), preserved neuronal structure, and good therapeutic efficacy. Curcumin‐NT also showed lower intensity (99.44), indicating a moderate protective response. Overall, the decreased intensity values in the Curcumin and WGX50‐T groups suggest better neuroprotection, reduced neuronal loss, highlighting their potential therapeutic value in AD management.

**TABLE 3:** Quantitative histopathological analysis of brain in neuroprotective efficacy of the nanoformulations.

Group	Mean
Control	125.925
WGX50	122.181
WGX50‐T	108.361
WGX50‐NT	122.644
Curcumin	92.416
Curcumin‐T	125.131
Curcumin‐NT	99.444

In histological analysis, WGX50‐NT (127.35), Curcumin‐T (123.97), and WGX50 (122.13) showed higher mean intensities compared to the control (116.65), indicating greater tissue presence and possible accumulation within brain regions. Curcumin (93.62) and Curcumin‐NT (102.94) showed relatively lower intensities, suggesting reduced distribution.

### 3.13 Fluorescence Patterns in Targeted and Non‐Targeted Rats

**FIGURE 15:**
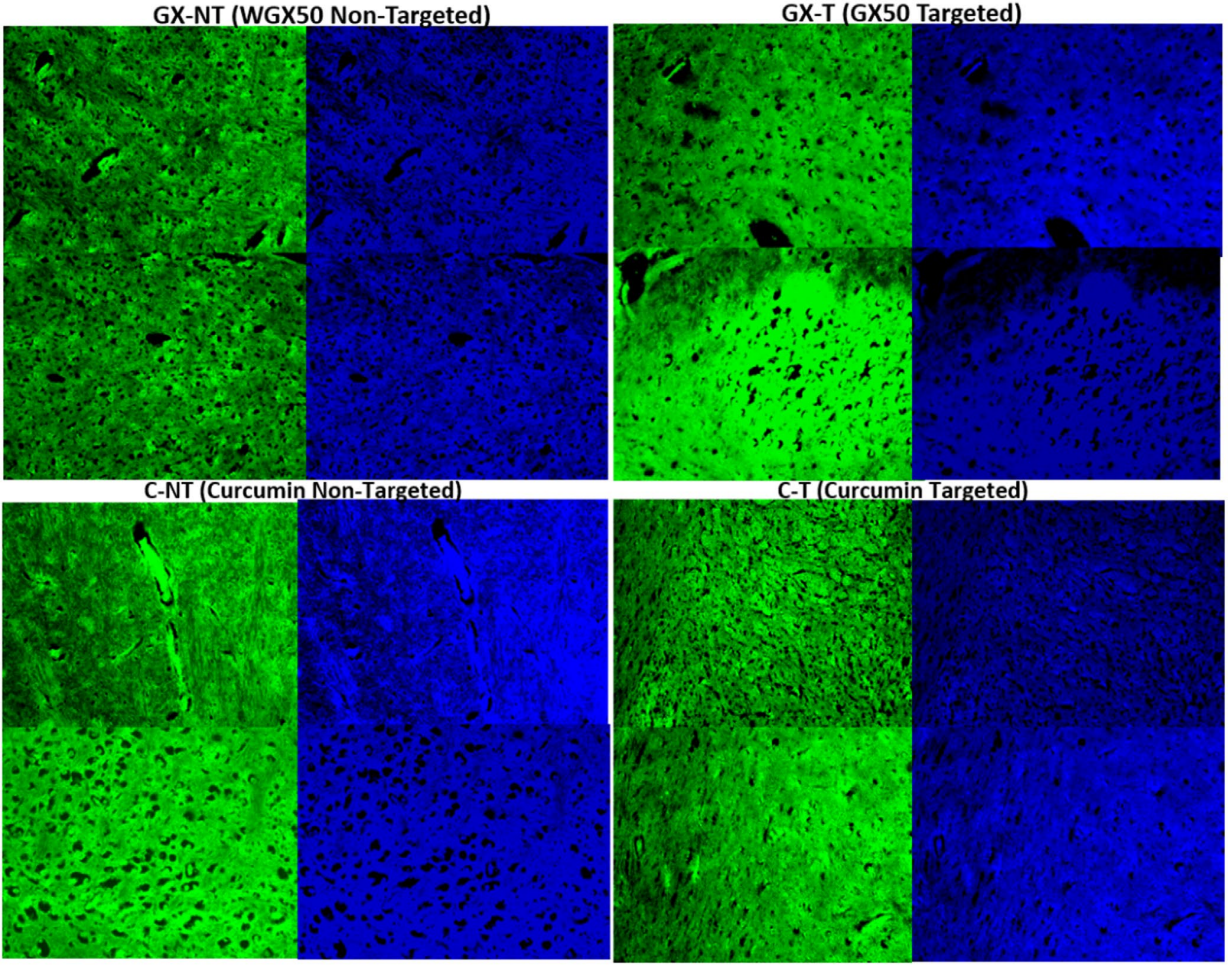
The fluorescence microscopy images of brain tissues of AD treated rats with Curcumin non‐targeted (C‐NT), Curcumin targeted (C‐T), WGX50‐NT, and WGX50‐T. The green fluorescence likely represents the presence of curcumin, while the blue fluorescence indicates cell nuclei stained with a counterstain (DAPI).

The differences in fluorescence patterns between the targeted and non‐targeted treatments suggest that targeted delivery (WGX50‐T and C‐T) is more effective at directing the therapeutic agents to specific brain regions (Figure 15). This targeted concentration could potentially lead to better therapeutic outcomes in AD‐treated rats by ensuring that higher concentrations of the therapeutic agents are available at the sites of action. The non‐targeted treatments (WGX50‐NT and C‐NT) show diffuse distribution of the therapeutic agents, which may result in less effective treatment due to lower concentrations in the target areas.

The brain tissues of AD‐treated rats with Curcumin‐NT, Curcumin‐T, WGX50‐NT, and WGX50‐T have been shown (Figure 15, Table 3). The brain tissues of treated rats with WGX50‐T (AuNPs‐WGX50‐Insluin) and WGX50‐NT (AuNPs‐WGX50), respectively, showed intense green fluorescence, suggesting a high concentration of the targeted molecule in the brain tissue.

Fluorescence microscopy data, which reflects the extent of BBB penetration, showed that WGX50‐T (65.95) and WGX50 (63.62) exhibited the highest fluorescence intensities, demonstrating brain uptake and efficient BBB crossing. Curcumin‐T (57.94) also showed higher fluorescence than control (46.26). The WGX50‐NT (43.95) and Curcumin‐NT (52.63) exhibited lower intensities, suggesting limited BBB permeability. Overall, the findings suggest that targeted formulations, particularly WGX50‐T and Curcumin‐T, enhanced BBB transport and brain accumulation compared to non‐targeted and free compounds (Table 4).

**TABLE 4:** Mean intensity values from histological analysis and fluorescence microscopy.

Histological analysis		Fluorescence microscopy
Compound/nanoconjugate	Mean intensity	Mean intensity
Control	116.649	46.258
WGX50	122.133	63.618
WGX50‐T	111	65.952
WGX50‐NT	127.348	43.951
Curcumin	93.624	46.909
Curcumin‐T	123.965	57.939
Curcumin‐NT	102.935	52.631

### 3.14 Expression Analysis of miRNA‐146a‐5p in a Rat Model of AD

The hsa‐miR‐146a‐5p is a microRNA that has been extensively implicated in AD development. To investigate the expression levels of hsa‐miR‐146a‐5p in the context of AD, serum samples were collected from albino rats ( *Rattus norvegicus* ) in which AD was experimentally induced. Quantitative PCR (qPCR) was performed using specific primers: Forward primer (5′‐TGCGCTGAGAACTGAATTCCAT‐3′) and reverse primer (5′ CCAGTGCAGGGTCCGAGGTATT‐3′). The qPCR analysis was conducted to quantify the expression levels of miRNA‐146a‐5p in a rat model of AD. The amplification plot provided the fluorescence data across different cycles for the target miRNA. The Ct (cycle threshold) values were determined by identifying the point where the fluorescence signal exceeded the threshold set above the baseline (Figure S2).

The ΔCt values were calculated by subtracting the Ct values of the housekeeping gene from the Ct values of miRNA‐146a‐5p for each sample.

### 3.15 Relative Expression Analysis

The ΔCt values indicate the relative expression levels of miRNA‐146a‐5p in comparison to the housekeeping gene. Lower ΔCt values correspond to higher expression levels of miRNA‐146a‐5p relative to the housekeeping gene, while higher ΔCt values suggest lower relative expression. Samples 7 to 12 exhibited negative ΔCt values, indicating that the expression levels of miRNA‐146a‐5p were higher than those of the housekeeping gene (Table 5). Notably, Sample 11 had the most negative ΔCt value (−7.0141), indicating the highest relative expression of miRNA‐146a‐5p. Samples 1 to 6 showed positive ΔCt values, suggesting lower relative expression levels of miRNA‐146a‐5p compared to the housekeeping gene. Among these, Sample 2 had the highest ΔCt value (3.858), indicating the lowest relative expression of miRNA‐146a‐5p.

**TABLE 5:** Ct values of miRNA‐146a‐5p for each sample.

Sample no. (treatment)	Ct (target gene)	Ct (housekeeping gene)	ΔCt = Ct (target)—Ct (housekeeping)
Sample 1 (Curcumin)	38.622517	35.796884	2.825633
Sample 2 (Curcumin)	40.0	36.142	3.858
Sample 3 (C‐NT)	37.458073	35.998	1.460073
Sample 4 (C‐NT)	38.195557	36.204	1.991557
Sample 5 (C‐T)	39.546535	36.110	3.436535
Sample 6 (C‐T)	40.0	39.457900	0.5421
Sample 7 (WGX50‐T)	37.959338	39.392	−1.432662
Sample 8 (WGX50‐NT)	39.457424	39.510	−0.052576
Sample 9 (WGX50‐T)	36.787786	39.421	−2.633214
Sample 10 (WGX50‐NT)	39.097746	39.470	−0.372254
Sample 11 (WGX50)	32.4809	39.495	−7.0141
Sample 12 (WGX50)	36.526928	39.532	−3.005072

Statistical analysis using one‐way ANOVA revealed significant differences in ΔCt values of hsa‐miR‐146a‐5p expression in treatment groups (*F* = 8.32, *p* = 0.011). The effect size (*η* ^2^ = 0.87) indicated a large treatment effect, suggesting that the type of formulation had a substantial influence on miRNA expression. Post hoc Tukey's test further confirmed that WGX50 and its targeted form (WGX50‐T) produced significantly lower ΔCt values, indicating stronger suppression of hsa‐miR‐146a‐5p expression.

The upregulation of miRNA‐146a‐5p in specific samples (7 to 12) could suggest its involvement in modulating neuroinflammation and immune responses in the AD model (Figures S1–S4). The observed expression patterns align with the hypothesis that miRNA‐146a‐5p plays a role in regulating inflammatory pathways in neurodegenerative diseases.

The target gene's and the housekeeping gene's Ct (cycle threshold) values, as well as the ΔCt value—the difference between the target gene's and the housekeeping gene's Ct values—are highlighted in the graph that shows the gene expression levels in AD for a particular sample.

## 4 Discussion

A widespread AD primarily damages memory performance and important brain functions [25]. AD stands as the most common type of dementia and reports this fact through the Alzheimer's Association (2022). Advanced age acts as a risk factor for AD development yet the disease should not be considered a natural consequence of aging. The progressive nature of AD symptoms necessitates careful monitoring of disease advancement [26, 27]. Researchers concentrate on disease slowness through their efforts because the medical community has yet to discover an effective treatment for AD. Scientific research continues to reevaluate the multiple factors that cause AD [28].

We designed a novel nanoformulation (AuNP‐WGX50‐insulin and AuNP‐curcumin‐insulin) for targeted drug delivery systems which represent a significant improvement in the treatment of AD by enhancing the delivery of therapeutic agents across the blood–brain barrier and directly to the affected brain regions. To our knowledge, this is the first study to combine WGX50 and curcumin on an insulin‐targeted AuNP scaffold, thereby integrating a dual‐drug strategy with receptor‐mediated BBB targeting for AD therapy. Compared with prior curcumin nanoparticle reports, our targeted nanoconjugates achieved markedly higher Aβ clearance and improved miRNA‐linked anti‐inflammatory responses, highlighting advantages in both delivery and pharmacodynamics. AuNP provide excellent drug loading and colloidal stability; cysteine capping allows stable insulin conjugation, enabling receptor‐mediated transcytosis across the BBB compared with non‐targeted formulations [29, 30, 31]. These systems can enhance the efficacy of drugs and reduce side effects by concentrating the treatment where it is needed most. Previous studies have developed nanoparticles that can be engineered to cross the blood–brain barrier and deliver drugs directly to the brain, potentially improving the treatment of AD [32, 33]. Targeted drug delivery systems for AD treatment using insulin is important in improving cognitive functions and modulate inflammation, a key component of AD pathology [34, 35].

The use of AuNP‐WGX50‐insulin (WGX50‐T) and AuNP‐curcumin‐insulin (curcumin‐T) nanoformulations for targeted delivery in AD rats represents an innovative approach to enhance the therapeutic efficacy of WGX50 and curcumin. These nanoformulations aim to improve drug delivery across the blood–brain barrier in AD treatment. Incorporating insulin, these systems facilitate the transport of attached WGX50 and curcumin into the brain, potentially increasing their concentration at the target site and thus improving their impact on AD pathology. In our study, insulin was selected as a targeting ligand to facilitate transport across the BBB [36, 37]. The endothelial cells in BBB express insulin receptors, which mediate transcytosis [38]. By conjugating insulin to the nanoparticle surface, the nanocarriers can exploit this natural transport mechanism to cross the BBB more efficiently. Previous studies have demonstrated that insulin‐modified nanoparticles show enhanced uptake into the brain through insulin receptor binding and internalisation pathways, thereby improving central nervous system drug delivery [39, 40]. Thus, the choice of insulin provides a rational, receptor‐mediated strategy for targeted delivery of therapeutic agents into the brain, enhancing both bioavailability and therapeutic efficacy in Alzheimer's disease models.

WGX50, derived from Zanthoxylum bungeanum Maxim, has been reported to inhibit the activation of JAK2/STAT3 and PI3K/AKT pathways by the induction of amyloid‐beta peptide for Alzheimer's disease (AD) [41]. This compound has shown anti‐inflammatory and antioxidant effects similar to those of *Zanthoxylum bungeanum Maxim*.

The quantitative histopathological analysis of Aβ plaque in brain sections has been given in Table 3 to evaluate the neuroprotective efficacy of different nanoformulations. The parameters including “Area” and “Mean intensity” represent the density of Aβ deposition. Higher mean intensity values correspond to greater Aβ aggregation [42], while reduced mean indicate low plaque burden, reflecting neuroprotection. The WGX50‐T (targeted WGX50 nanoformulation) shows a good reduction in mean intensity (108.36) compared to the control (125.93, 2.45) and non‐targeted formulation (WGX50‐NT, 122.64, 2.456). This suggests that WGX50‐T effectively reduces the density of Aβ plaques in the brain. The targeted delivery likely enhanced its brain uptake, improving the clearance of Aβ aggregation (Figures 14 and 15). This suggests that the non‐targeted approach may distribute the therapeutic agent more broadly, which could be less effective at the sites of pathology.

The histological and fluorescence intensities data (Table 4) indicate that targeted formulations markedly enhance brain accumulation compared with their non‐targeted. These patterns are consistent with receptor‐mediated transcytosis improving nanoparticle transport across the BBB; ligand functionalization (insulin) has repeatedly been shown to increase brain uptake by engaging endothelial receptors and triggering endocytic transport [43, 44]. The relatively high histological intensities of WGX50 and WGX50‐NT (122.13 and 127.35, respectively) suggest that favourable physicochemical properties of the WGX50 can also promote tissue retention independently of active targeting—a phenomenon where nanoparticle composition and colloidal stability drive passive accumulation in brain tissue [45].

The differences in staining intensity and distribution among the brain sections in Figure 15 highlight the potential benefits of targeted drug delivery systems in enhancing the therapeutic effects of WGX50 on AD pathology (Figure 15). These findings highlight the importance of targeted drug delivery, which could lead to more effective treatment outcomes. Further studies are needed to quantify and explore the long‐term effects of these treatments on brain health.

Curcumin, known for its neuroprotective effects, is a promising candidate in neurodegenerative disorders due to its ability to modulate different signalling pathways involved in their development including nuclear factor‐erythroid 2‐related factor 2 (Nrf2), serine/threonine kinase AKT, and transcription factor nuclear factor‐kB (NF‐kB) [17].

This targeted approach enhances the therapeutic effects of curcumin by ensuring higher local concentrations at the sites of pathology, leading to more effective reduction of AD‐related markers [46, 47]. The comparison between Curcumin‐NT and Curcumin‐T groups highlights the potential benefits of targeted drug delivery systems in enhancing the efficacy of curcumin treatment for AD [48]. These systems can improve the bioavailability and specificity of therapeutic agents, which is vital for treating complex diseases like AD [49]. Some conventional nanocarriers or solid lipid NPs have improved curcumin's stability; they still face limitations in optimal brain delivery. For example, vesicular and liposomal curcumin systems show improved absorption and extended circulation time compared to free curcumin, but the brain penetration is still poor [50]. However, AuNP‐based curcumin delivery may have some advantages: (1) strong surface plasmon resonance enables efficient functionalization, enhancing cellular uptake [51]. (2) The Au–S (gold–thiol) anchoring strategy may increase the chemical stability of the curcumin (or linker‐curcumin) compared to lipid or polymer, which may have leakage or degradation issues [52]. (3) AuNPs can readily be engineered with targeting ligands (brain‐targeting moieties), enhancing BBB receptor‐mediated uptake for neuroprotective outcomes. Thus, the AuNP approach offers a novel delivery advantage beyond liposomal or polymeric nanoformulations particularly in neuroprotection.

The microRNA‐146a‐5p (miRNA‐146a‐5p) is involved in the pathogenesis of AD. It functions as a critical negative feedback regulator of the NF‐κB pathway, as a key player in inflammatory responses [53]. In AD, miRNA‐146a‐5p could be a potential therapeutic target for modulating inflammatory responses and treatment. The analysis of the Ct values suggests that targeted drug delivery systems (Table 3) may be more effective in regulating miRNA‐146a‐5p expression, which could have implications for AD treatment. The Ct values (Table 3) reflect the relative expression levels of miRNA‐146a‐5p across different groups. A lower ΔCt value suggests higher expression of miRNA‐146a‐5p. Comparing the ΔCt values of curcumin treated groups (Sample 1: 2.825633, Sample 2: 3.858) with non‐targeted (C‐NT) and targeted (C‐T) curcumin, the targeted curcumin treatment (Sample 6: 0.5421) results in a lower ΔCt value, suggesting a downregulation of miRNA‐146a‐5p expression. This suggests that targeted delivery of curcumin may be more effective in modulating miRNA‐146a‐5p levels, leading to better therapeutic outcomes in AD. The DEX treatment has been shown to downregulate inflammation‐related miRNAs, including miR‐147‐3p, miR‐146a‐5p, miR‐146b‐5p, and miR‐34a‐5p, compared to inflamed untreated controls [54]. This shows that miRNA‐146a‐5p could be a potential therapeutic target for modulating inflammatory responses in AD patients.

The ΔCt values revealed a statistically significant effect of treatment on hsa‐miR‐146a‐5p expression (ANOVA *p* < 0.05) with a large effect size, demonstrating that treatment type accounts for a considerable portion of the observed variance. Post hoc comparisons indicate that the WGX50 groups (both targeted and non‐targeted) vary from curcumin‐based groups, consistent with the markedly lower ΔCt values observed for WGX50 samples. Thus, in our dataset, WGX50 treatment is associated with relatively elevated miR‐146a‐5p levels compared with curcumin treatments, while curcumin formulations tend to show increased ΔCt (lower miR‐146a‐5p expression).

Biologically, miR‐146a has been repeatedly implicated in innate immune signalling and neuroinflammation in AD and related neurodegenerative disorders [36, 55]. Several reports show dysregulation of miR‐146a in the AD brain and in models of amyloid pathology, and the miRNA can modulate Toll‐like receptor/NF‐κB pathways and cytokine signalling that influence Aβ pathology and neuroinflammation [56]. These prior studies suggest that changes in miR‐146a expression reflect modulation of neuroinflammatory responses that may be relevant to therapeutic outcomes. Our observation that curcumin formulations reduce miR‐146a (higher ΔCt → lower expression) is consistent with previous reports that curcumin can downregulate miR‐146a and dampen inflammatory signalling in AD models. Conversely, the apparent upregulation of miR‐146a with WGX50 may reflect compound‐specific engagement of immune pathways or an early compensatory response; WGX50 (gx‐50) has previously been reported to exert neuroprotective and anti‐inflammatory effects in AD models but may also modulate innate immune signalling in a context‐dependent manner [57, 58]. Given the complex and sometimes bidirectional role of miR‐146a in neuroinflammation, these differential effects between WGX50 and curcumin are biologically reasonable and need further studies.

One important limitation of this study is the small number of animals included in some groups, as the present work was conceived as a pilot study to evaluate feasibility and generate preliminary data. Although these small groups yielded useful observations, the statistical power is limited, and findings need to be interpreted cautiously. In future studies, larger and statistically powered cohorts will be included to further strengthen these results.

## 5 Conclusion

The use of targeted drug delivery systems, specifically AuNP‐WGX50‐insulin and AuNP‐curcumin‐insulin nanoformulations, significantly impacts the reduction of Aβ plaque levels in AD induced SD rats. The immunohistochemical assessment reveals that both WGX50‐NT and WGX50‐T treatments contribute to a substantial decrease in Aβ oligomers, highlighting their potential to cross the blood–brain barrier and enhance cognitive function. Notably, the targeted therapy (WGX50‐T) proves a more obvious effect, which is crucial for protecting neurons from damage. The fluorescence microscopy analysis also confirms that targeted delivery (WGX50‐T and C‐T) is more effective at directing therapeutic agents to specific brain regions, leading to higher local concentrations for better therapeutic outcomes. The analysis of miRNA‐146a‐5p expression levels provides therapeutic effects of these treatments. The upregulation of miRNA‐146a‐5p in specific rat groups suggests its involvement in modulating neuroinflammation and immune responses in the AD model. This aligns with the hypothesis that miRNA‐146a‐5p plays a role in the regulation of inflammatory pathways in neurodegenerative diseases. These findings collectively suggest that targeted drug delivery systems, utilising insulin as a carrier, are promising for the treatment of AD. The modulation of miRNA‐146a‐5p expression levels highlights the potential of these nanoformulations to improve cognitive function and protect neurons in AD, warranting further investigation into their clinical application.

## Author Contributions

Concept: M.S.L., M.T.K., and D.W. Methodology: M.M. and A.B. Funding: K.M. Writing – original draft: M.T.K. and A.B. Writing – review and editing: K.M., M.S.L., and M.T.K.

## Ethics Statement

Ethical approval was taken from the Institutional Ethical Board of the Institute of Molecular Biology and Biotechnology (IMBB), the University of Lahore (2024/IMBB/UOL).

## Conflicts of Interest

The authors declare no conflicts of interest.

## Supporting information


**Data S1:** jcmm71045‐sup‐0001‐DataS1.zip.

## Data Availability

The data that supports the findings of this study are available in the Supporting Information of this article.
