# Supplementary material for: Targeted Nanodelivery of WGX50 and Curcumin via Gold Nanoparticles for Alzheimer's Therapy
Source: J Cell Mol Med. 2026 Feb 6;30(3):e71045. doi: 10.1111/jcmm.71045 (PMC12877722; doi:10.1111/jcmm.71045)
Supplement: Supplementary file 1 — Data S1: jcmm71045‐sup‐0001‐DataS1.zip. [file JCMM-30-e71045-s001.zip › Supplementary Tables.docx]

**Table. S1** Shows the elements and their weight % inside the AuNPs.

| **Element** | **Weight %** | **Atomic %** | **Error %** |
| --- | --- | --- | --- |
| C K | 17.33 | 24.03 | 7.15 |
| N K | 21.59 | 25.68 | 9.55 |
| O K | 46.54 | 48.47 | 9.73 |
| Na K | 0.47 | 0.34 | 22.32 |
| Mg K | 0.23 | 0.16 | 20.46 |
| Al K | 0.13 | 0.08 | 24.63 |
| Au M | 13.46 | 1.14 | 7.07 |
| Ca K | 0.25 | 0.1 | 19.11 |

**Table S2.** Shows the elements and their weight % inside the cysteine-capped AuNPs.

| **Element** | **Weight %** | **Atomic %** | **Error %** |
| --- | --- | --- | --- |
| C K | 12.88 | 35.36 | 9.75 |
| O K | 14.46 | 29.82 | 9.99 |
| Ni L | 1.2 | 0.67 | 8.44 |
| Na K | 14.29 | 20.5 | 8.51 |
| Mg K | 0.58 | 0.78 | 14.48 |
| Au M | 52.13 | 8.73 | 3.06 |
| Cl K | 4.34 | 4.04 | 7.51 |
| Ca K | 0.12 | 0.1 | 62.31 |
