# Supplementary figures and images for: Targeted Nanodelivery of WGX50 and Curcumin via Gold Nanoparticles for Alzheimer's Therapy
Source: J Cell Mol Med. 2026 Feb 6;30(3):e71045. doi: 10.1111/jcmm.71045 (PMC12877722; doi:10.1111/jcmm.71045)

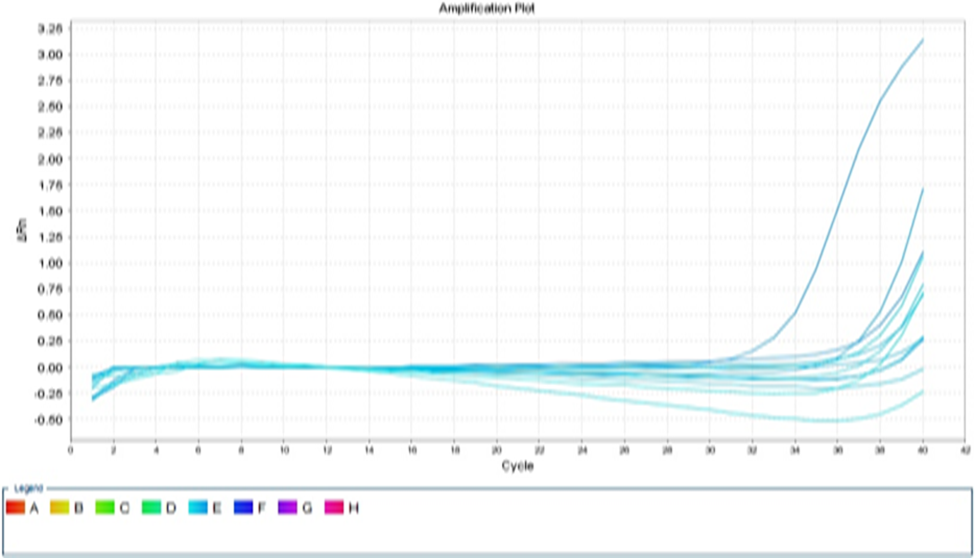

Supplement: Supplementary file 1 — Data S1: jcmm71045‐sup‐0001‐DataS1.zip. [file JCMM-30-e71045-s001.zip › Figure_S2.tif]

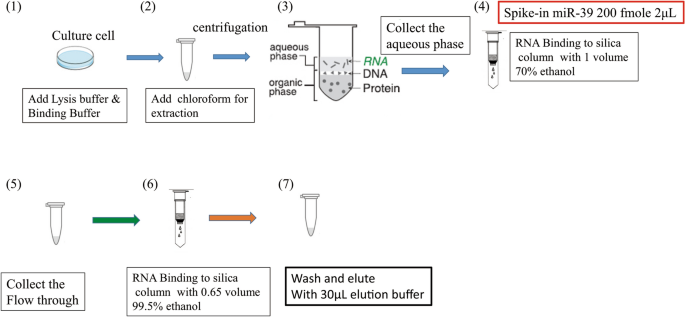

Supplement: Supplementary file 1 — Data S1: jcmm71045‐sup‐0001‐DataS1.zip. [file JCMM-30-e71045-s001.zip › Figure_S1.tif]
