# Supplementary material for: Targeted Nanodelivery of WGX50 and Curcumin via Gold Nanoparticles for Alzheimer's Therapy
Source: J Cell Mol Med. 2026 Feb 6;30(3):e71045. doi: 10.1111/jcmm.71045 (PMC12877722; doi:10.1111/jcmm.71045)
Supplement: Supplementary file 1 — Data S1: jcmm71045‐sup‐0001‐DataS1.zip. [file JCMM-30-e71045-s001.zip › S4_Revised Trial images for comparison.zip!/All for comparison/Groups information.docx]

| 1. | AD rats treated with WGX50-NT |
| --- | --- |
| 2. | AD rats treated with Curcumin-T |
| 3. | AD rats treated with Curcumin-NT |
| 4. | AD rats treated with WGX50-T |
| 5. | AD rats treated with WGX50 |
| 6. | Healthy rats not treated |
